# Federated deep learning for stroke detection: a privacy-preserving, explainable CNN-LSTM framework

**DOI:** 10.3389/fnins.2026.1847277

**Published:** 2026-09-21

**Authors:** Reeta Samuel, Thanapal Pandi

**Affiliations:** School of Computer Science Engineering and Information Systems, Vellore Institute of Technology, Vellore, India

**Keywords:** brain stroke, CNN, deep learning, federated learning, LSTM, MRI

## Abstract

Strict privacy regulations in healthcare limit the centralized sharing of medical imaging data, creating challenges for developing robust and generalizable diagnostic models. Federated learning (FL) offers a privacy-preserving paradigm in which participating institutions collaboratively train a global model without directly exchanging patient images. This study presents a federated CNN-LSTM framework for privacy-preserving brain stroke classification from MRI scans, designed to learn discriminative spatial and sequential representations while retaining training data at the local client level. The proposed framework employs a CNN for hierarchical spatial feature extraction and an LSTM to model sequential dependencies among extracted features, followed by federated averaging (FedAvg) to construct the global model from local updates. Experimental evaluation on a private MRI dataset comprising 3,125 scans distributed across three non-IID clients enabled assessment under heterogeneous data distributions. The proposed federated framework achieved an accuracy of 95.20%, compared to 95.95% for centralized training, demonstrating that privacy-preserving decentralized training can achieve competitive diagnostic performance with only a marginal reduction in accuracy. Furthermore, Grad-CAM was employed to provide visual explanations of the model’s predictions by highlighting the image regions that contribute to stroke classification. In five-fold cross-validation, the Federated CNN-LSTM model achieved an average accuracy of 92.10% ± 1.50%, indicating consistent performance across folds. The findings demonstrate the feasibility of combining federated learning, hybrid CNN-LSTM representation learning, and model interpretability for privacy-preserving MRI-based stroke classification, while highlighting the need for multi-center validation and larger federated deployments before clinical translation.

## 1 Introduction

Stroke is a significant global health problem that frequently results in unilateral weakness among survivors. This impairment considerably affects their independence in daily life, particularly when it interferes with their capacity to undertake tasks requiring delicate hand movements (Rungsirisilp and Wongsawat, 2022). Strokes occur either because of a blood vessel bursting, known as a hemorrhagic event, or because of a vessel blockage, referred to as an ischemic event. These two types are diagnosed using imaging methods, including computed tomography (CT) and magnetic resonance imaging (MRI) (Mousavi and Majedi, 2025). Strokes or cerebrovascular accidents occur when there is an interruption or reduction in the flow of blood to the brain, leading to a severe shortage of oxygen and nutrients delivered to brain cells (Tanveer et al., 2024). Significant progress in the diagnosis of brain disorders has been achieved using deep learning (DL) approaches. Machine learning (ML) methods have demonstrated outstanding results in tasks requiring the categorization of brain networks (Zhang et al., 2023).

Accurate medical image classification is a critical element in healthcare, supporting various tasks, including disease diagnosis, treatment planning, and drug development. The automation of this process is critical to enhancing patient-focused care, especially regarding the detection of irregularities in images obtained from MRI, CT, and X-ray equipment. These imaging methods provide detailed visualizations of internal structures, thereby facilitating physicians’ judgments and surgical preparation (Srinivasu et al., 2024). Convolutional neural networks (CNNs) have brought image analysis to the forefront of deep learning, revolutionizing computer vision.

Medical image processing professionals have used these developments in several applications, such as registration, diagnosis, and forecasting patient outcomes (He et al., 2016). A subset of machine learning, deep learning models, has achieved notable outcomes in medical image analysis by employing deep neural networks to process significant quantities of annotated data and then discover primary shapes and associations within the data (Abbasi et al., 2023).

One of the main advantages of deep learning is its capacity to automatically identify and extract advanced characteristics from unprocessed information, thereby eliminating the need to collect physical characteristics, unlike conventional machine learning methods that require features as input. Deep learning has demonstrated significant achievements in a range of medical tomography tasks, including image creation of images, lesion segmentation, and early illness detection (Suganyadevi et al., 2022). Techniques based on convolutional neural networks (CNNs), such as VGG, ResNet, and U-Net, are often employed for accurate stroke lesion identification and segmentation, thereby reducing reliance on manual feature extraction. Despite ongoing challenges with interpretability and data integration, improvements in ML and DL are expanding the capabilities of stroke diagnosis (Lövblad and Baird, 2010).

Convolutional neural networks (CNNs), a type of DL, have significantly impacted the discipline of exploring medical images by successfully identifying patterns and categorizing images. Detailed anatomical visualizations are vital for healthcare provision. Sharing sensitive medical data in a traditional centralized learning environment poses substantial privacy and security threats (Frik et al., 2019), and brain imaging techniques, such as CT and MRI scans, are essential for detecting abnormalities in the brain. A non-invasive method is particularly useful for studying central nervous system (CNS) disorders. Detailed slice-by-slice imaging capabilities enable doctors to visualize brain structures with high sensitivity, facilitating accurate diagnosis and patient communication. This imaging technique surpasses CT scans by providing superior image contrast and real-time visualization of brain anatomy during scans (Londhe, 2017).

Collaborative machine learning is facilitated by federated learning, which allows model training on distributed local data across numerous devices without the need for centralized storage. This approach prioritizes data privacy by storing sensitive information on individual devices (Li et al., 2021). The central server oversees the learning process and generates a highly effective model that integrates all updates from the individual devices. It also distributes pre-trained models to devices to speed up learning, and each device sends its acquired knowledge to the global model of the central server (Subashini et al., 2016). Federated transfer learning provides a solution to the challenges of information integration with privacy in health imaging, particularly in the classification of brain tumors. The merging of federated and transfer learning enables deep convolutional neural networks (CNNs) to be trained on decentralized, sensitive medical data without the need for direct data sharing (Soumik and Hossain, 2020). Using pre-trained weights accelerates model convergence and enhances generalization capabilities by sharing knowledge across different datasets. Consequently, FTL improves privacy, scalability, and fault tolerance in medical applications (Appasami and Savarimuthu, 2025).

The key findings of this study are as follows.

- A private brain stroke MRI dataset was curated and pre-processed from hospital records, with raw DICOM files standardized and data augmentation techniques, such as rotation, scaling, and intensity variations, applied to enhance model generalization, reduce overfitting, and optimize performance for deep learning applications.
- A specialized CNN-LSTM model was developed to accurately classify strokes by capturing intricate spatial features.
- Successfully deployed a hybrid model within a federated learning (FL) environment, enabling decentralized training without compromising sensitive patient data.
- Integrated Grad-CAM analysis can visually highlight stroke-affected regions, providing crucial interpretability to assist physicians in clinical decision-making.
- Five-fold cross-validation is incorporated to rigorously assess the robustness, consistency, and generalizability of the proposed Federated CNN-LSTM model across multiple data partitions.
- Extensive evaluation demonstrated that the federated CNN-LSTM achieved state-of-the-art performance metrics (accuracy, precision, recall, and F1-score) with only a marginal trade-off compared to centralized training.

## 2 Related works

Recent studies have proposed various privacy-preserving federated learning frameworks for the Internet of Things (IoT), primarily by leveraging homomorphic cryptosystems. However, these frameworks face significant challenges, including high communication costs and failure rates, as well as difficulties in discerning and using high-quality data. Y. Chen et al. proposed the PFLLF framework, which utilizes the multi-key EC-ElGamal cryptosystem (MEEC) and federated sum optimization algorithm (FSOA) to address these issues, achieving reduced communication costs, enhanced security, and improved efficiency. The key techniques are MEEC for multi-key collaborative computing and FSOA for optimal training data acquisition. The performance evaluation of the framework demonstrates its superiority over existing solutions in terms of security, practicality, and efficiency. The limitations of existing frameworks include limited suitability for IoT applications, high communication costs, and suboptimal data quality, which the PFLLF framework aims to overcome. Future research directions include parallel computing and incentive mechanisms for privacy-preserving federated learning (Chen et al., 2024).

Samuel and Pandi (2025) introduced a voting classifier that integrates models to predict by selecting the class with the highest probability. This method is used to predict outcomes, such as election results. The weighted voting ensemble is considered more precise and reliable than hard voting because it considers the confidence of each base classifier, whereas hard voting counts the recognition of each model by the key classifiers. This technique is straightforward and can enhance the performance of a machine learning model in classification and regression tasks.

A. Ghosh and P. Krishnamoorthy proposed a novel federated learning framework, Federated Average Knowledge Distilled Mutual Conditional Learning (FedADC), for efficient waste classification. FedADC combines federated learning with knowledge distillation and mutual conditional learning, achieving enhanced model accuracy and adaptability. This study explored numerous ML and DL models, hyperparameter tuning procedures (grid and random searches), data augmentation, and denoising strategies. The results demonstrate that FedADC with Grid Search achieved superior accuracy, with DenseNet201 and ResNet152 exhibiting notable performance. This study demonstrates how FL can be utilized in cooperative model guidance that protects privacy and provides insights for applications beyond waste classification, including healthcare and environmental preservation (Ghosh and Krishnamoorthy, 2024).

Divya et al. proposed a convolutional neural network (CNN) model for skin disease classification using medical imaging while sustaining data isolation through FL. The typical achieved a median precision of 86% and a recall of 81% for seven skin disease groups. When combined with federated learning, the model obtained average accuracies of 82.421%, 87.262%, and 93.253% for several amounts of clients (500, 1000, and 2000 users). The proposed approach shows promise for effective skin disease categorization while maintaining patient data confidentiality. Future work will aim to expand the proposed model’s application, integrate IoT sensors, and support individual remote healthcare systems (Divya, Anand and Sharma, 2024).

Initially, Singh proposed utilizing a custom network before implementing a transfer learning approach with MobileNetV2, Xception, EfficientNetB7, ResNet152V2, and DenseNet201 models. MobileNetV2 achieved a higher accuracy of 96%. The system’s automated live trash-sorting system aims to boost recycling percentages. Previous research on waste categorization has focused on environmental issues resulting from household and medical waste. An earlier study on waste classification focused on the problems that household and medical waste cause to the environment (Singh et al., 2021).

Stephanie et al. (2022) proposed a deep neural network that utilizes differential privacy and transfer learning to generate a local model with minimal loss and enhanced efficiency. A DL structure using variance confidentiality was proposed by Abadi et al. (2016), which incorporates Laplacian noise into gradients. Xiang et al. (2016) noted that incorporating sound into the descent calculation enhances data privacy, but this approach significantly diminishes the model’s accuracy. Wang et al. (2024) proposed a trustworthy federated learning framework that incorporates a multimodal fine-grained model optimization technique and adaptive differential privacy protocols. Despite the measures taken, the image could still potentially compromise the partial privacy of the image (Aono et al., 2017). Qi et al. proposed the FedCRLD framework, which stands for Federated Contrastive Re-Localization Distillation, to improve medicinal segmentation precision around diverse regions and cycles. By effectively delocalizing server representations to clients, FedCRLD enables accurate segmentation of cardiac magnetic resonance (CMR) images. Notably, FedCRLD is the primary background for facilitating cross-region and cross-cycle CMR segmentation using FL, thereby advancing the field of medical image analysis while preserving data privacy (Qi et al., 2022).

An explainable federated learning framework for classifying brain tumors from MRI images was created by Mastoi et al. In order to facilitate cooperative training while maintaining the decentralization of medical data, the study combined GoogLeNet with federated learning. To see the areas affecting the model’s predictions, Grad-CAM and saliency maps were used. The system demonstrated the possibility of merging FL and XAI for interpretable and privacy-preserving medical diagnosis, achieving about 94% accuracy with 10 clients and 50 communication rounds (Mastoi et al., 2025).

Mu et al. addressed privacy and interpretability issues in remote healthcare data by proposing an explainable federated medical image analysis platform. In addition to using blockchain to increase security and trust, the work integrates explainability, causal learning, and federated learning. The work shows that explainability can be added to federated medical image analysis to improve the transparency and clinical significance of decentralized AI conclusions (Mu et al., 2024).

Agrawal et al. (2025) introduced U-FDL-PPE, a federated deep-learning framework that blends Grad-CAM-based explainability with privacy-preserving federated learning for medical image categorization. Chest X-ray images were used to train MobileNetV2 across dispersed healthcare facilities, and Grad-CAM produced heatmaps that displayed the image regions that made predictions. The study demonstrates how XAI enhances the transparency and clinical interpretability of AI predictions while FL can safeguard patient data.

## 3 Proposed methodology

This study utilized a three-stage methodology to identify brain strokes (BS). The initial step of data preparation entailed converting DICOM images to JPG format, assigning a stroke label to each image, and subsequently partitioning the images into three distinct subsets: training, testing, and validation sets. Model development and evaluation involved training and assessing a hybrid CNN-LSTM model to achieve the highest possible accuracy, with its performance being assessed using a test set. A central server and client site were established to implement federated learning, with pre-processed data used at the client side to train the model, and the results were compared to select the global model in the federated approach.

This study investigated the purpose of FL to accurately diagnose brain strokes from MRI scans while ensuring the confidentiality of the data involved. A hybrid model was created by combining CNN and LSTM networks to efficiently classify strokes. The hybrid model was subsequently integrated into a framework for distributed training using FL. This study confirms that a hybrid model can be effectively used in FL without substantial performance degradation, facilitating the development of robust medical-image classifiers despite limitations in data privacy. This study employed a sophisticated mixed-method model within a federated learning environment for the classification of brain strokes using magnetic resonance imaging (MRI). Figure 1 shows a graphical illustration of the proposed research. Figure 2 depicts a hybrid CNN–LSTM framework combined with FL to classify brain strokes using MRI data. Initially, spatial features are extracted and reduced in size through convolutional and pooling layers. These features are then flattened and fed into stacked LSTM layers to identify sequential patterns. The model is trained locally on various clients, with their learned weights combined using the FedAvg algorithm to create a global model. This process is repeated over several iterations to enhance the accuracy of classification to distinguish between stroke and normal cases.

**FIGURE 1:**
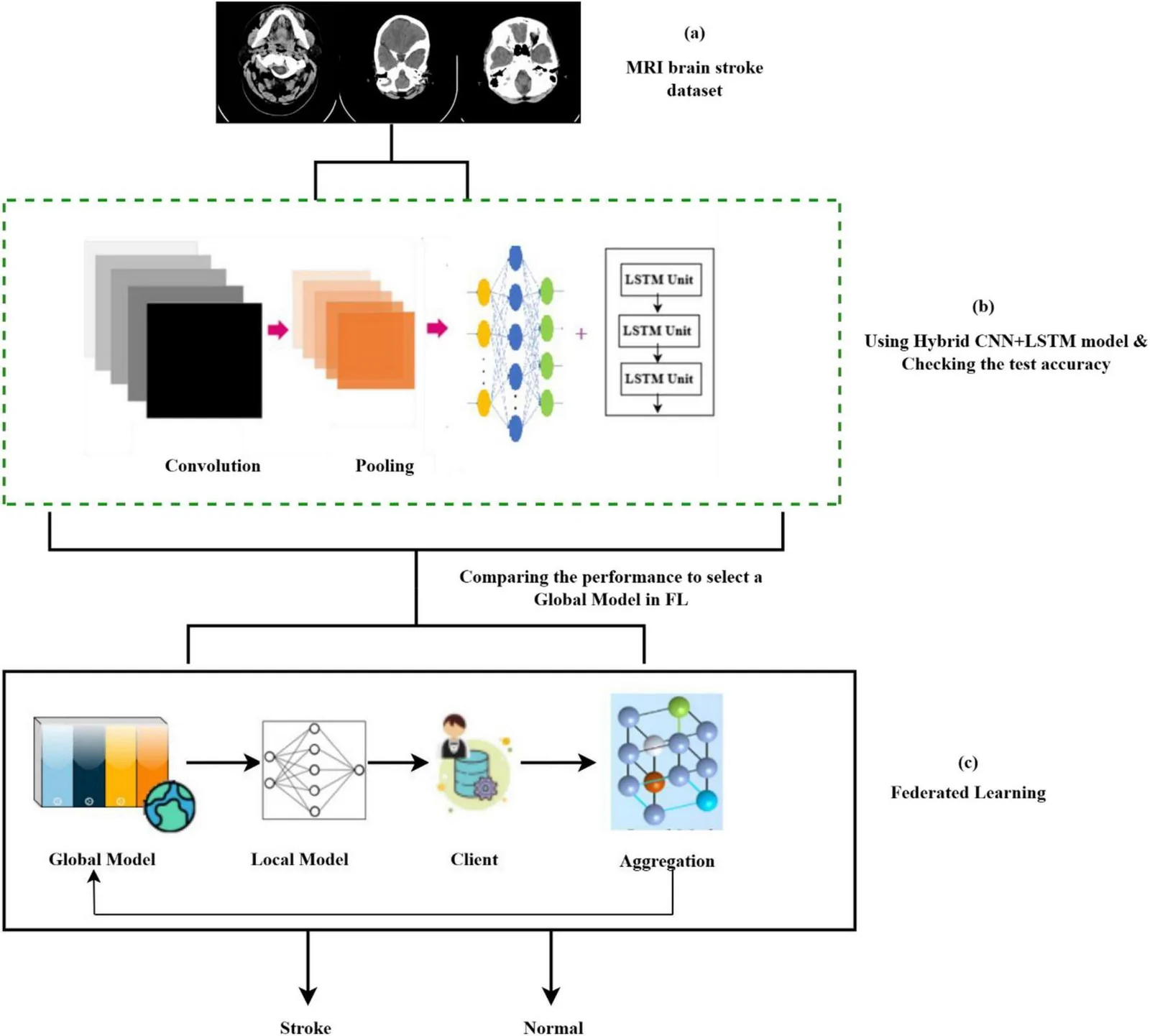
The suggested study’s visualization for identifying brain strokes in MRI datasets. **(a)** The dataset of MRI brain strokes. **(b)** The best methods for identifying brain stroke were the hybrid CNN and LSTM models. **(c)** Built a global model employing a hybrid model in FL, deployed it to local devices, returned the updated weights, and then used test results to evaluate the effectiveness for stroke identification.

**FIGURE 2:**
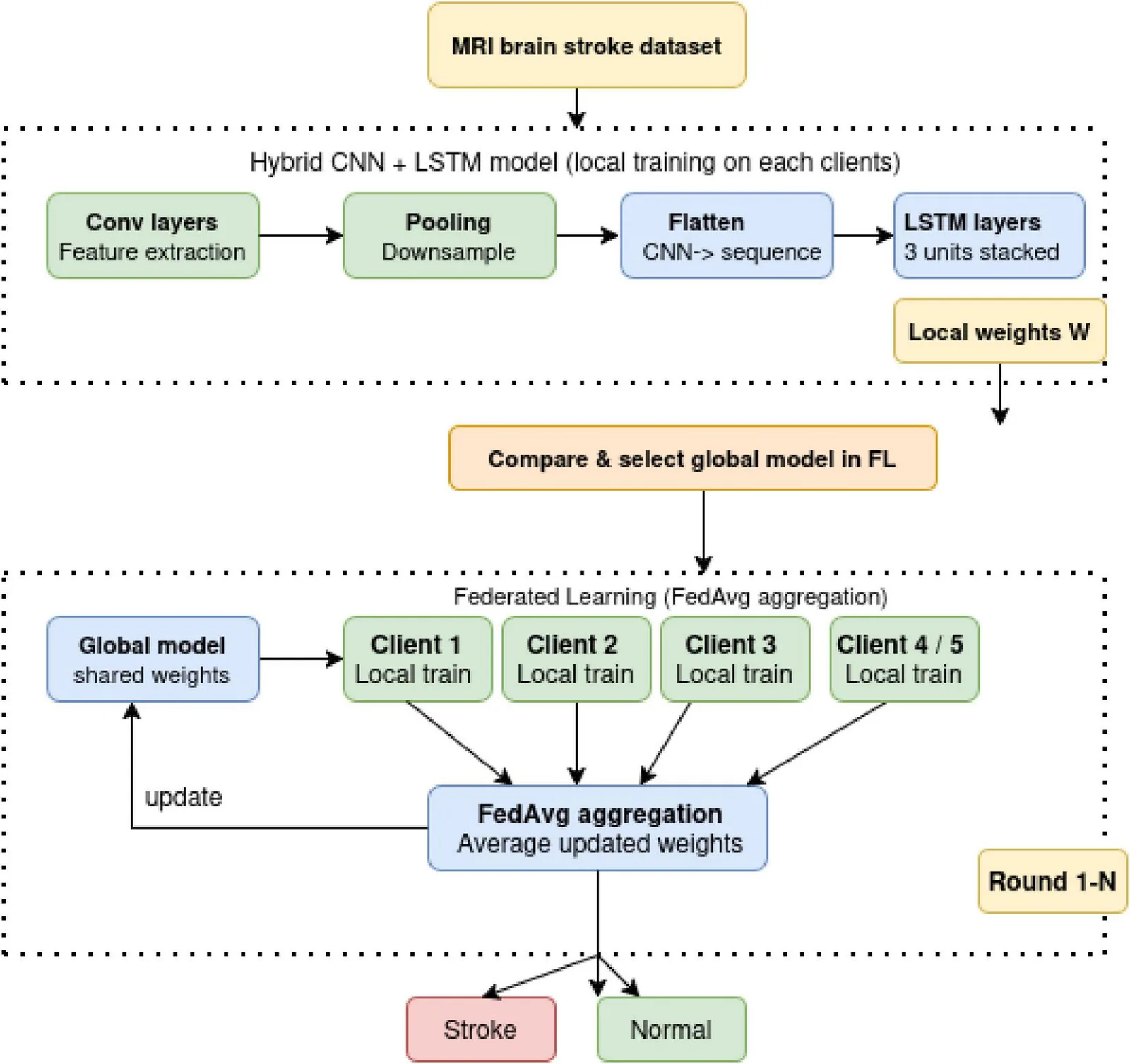
Hybrid CNN–LSTM-based Federated Learning framework for brain stroke classification using MRI images with FedAvg aggregation.

### 3.1 Data description

A private dataset containing MRI scans of stroke patients was obtained from KC Multispecialty Hospital in Chennai, India. The methods and experiments performed were purely computational and did not involve any direct human subjects. Patient confidentiality is of utmost importance. All procedures were conducted in accordance with the applicable guidelines and regulations. The KC Multispecialty Hospital, Chennai, India, approved all experimental protocols. The dataset consisted of MRI images used for surgical planning in brain stroke treatment, along with corresponding clinical data that featured unique patient identification numbers. This study employed a private brain stroke dataset consisting of 3125 MRI images for stroke classification. The dataset comprised two classes: it contained 1551 normal images and 1574 stroke images. The images were divided into three distinct groups, with 70% allocated for training, 10% for validation, and 20% reserved for testing. Figure 3 shows the BS dataset description. Table 1 shows the distribution of the brain stroke dataset.

**FIGURE 3:**
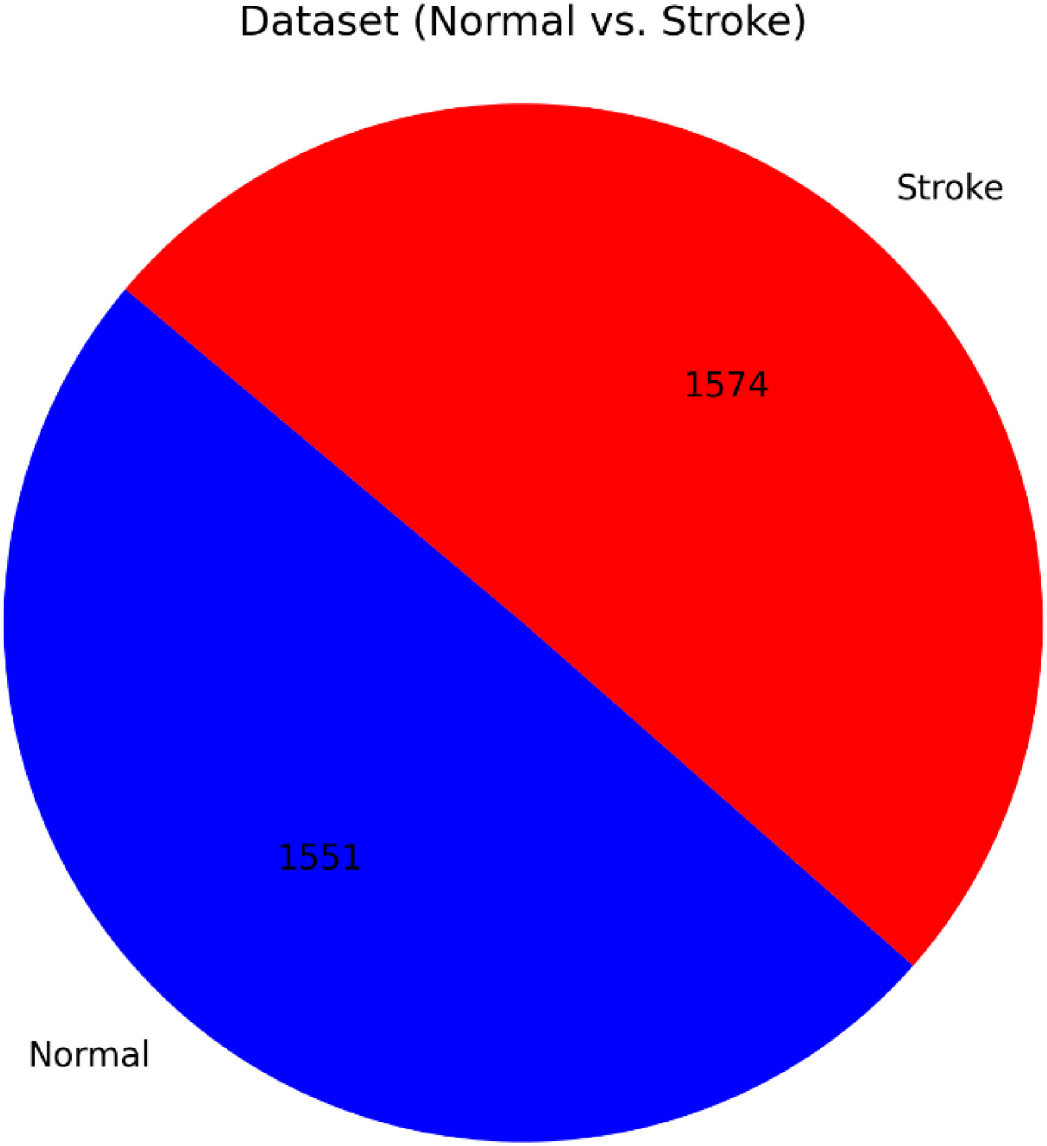
Dataset description.

**TABLE 1:** Brain stroke MRI dataset distribution.

Dataset	Total Images	Training set	Testing set	Validation set
Brain Stroke	3125 (Normal –1,551, Stroke –1,574)	2,188	625	312

### 3.2 Data pre-processing

Data pre-processing phases comprise this as one of their components. The DICOM images were first converted to JPG format and saved in the desired path to facilitate their handling and processing. Subsequently, a median filter was applied to the images to effectively reduce noise and enhance the overall image quality, thereby preparing them for further analysis and modeling. Median filters are often used as a pre-processing step to improve image quality before applying other image processing techniques, such as segmentation or feature extraction. The input images were then categorized and labeled as either “Stroke” or “Normal” based on whether a stroke was present or absent in the image. A sample of the prepared data is shown in Figure 4. Figure 5 shows the application of data augmentation techniques to an MRI brain image during pre-processing. The original image undergoes transformations into several variations (Aug 1–Aug 5) through processes such as rotation, minor scaling, translation, and changes in intensity. These augmentations enhance the diversity of the dataset, aiding the model in learning robust features that reduce overfitting and improve classification performance.

**FIGURE 4:**
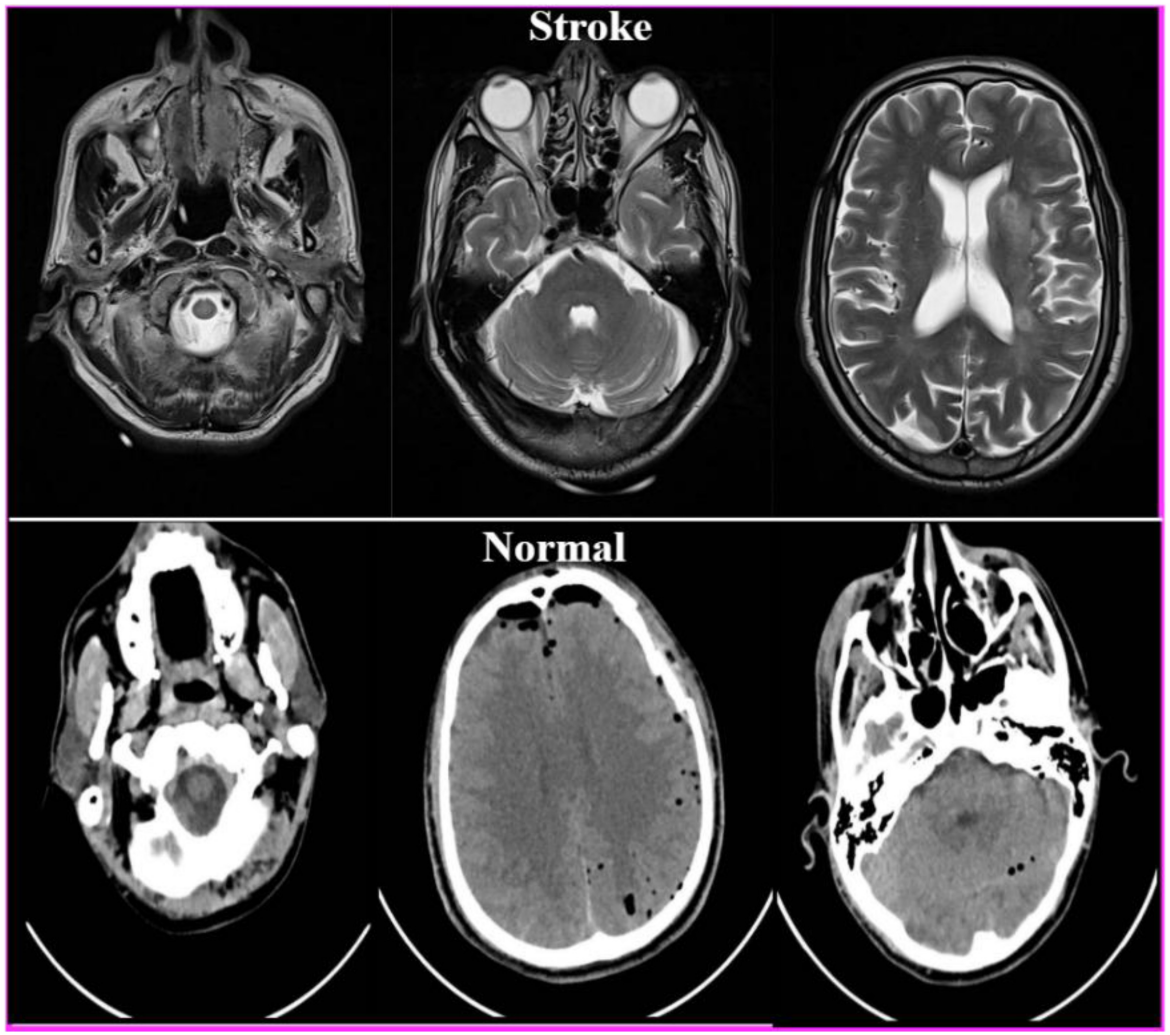
Sample images of the brain stroke dataset.

**FIGURE 5:**
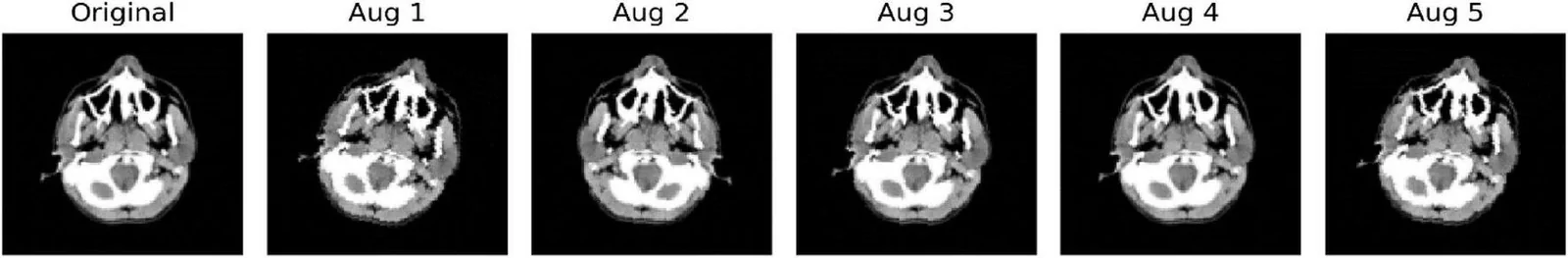
Brain MRI augmentation showing original and transformed images for improved generalization.

### 3.3 Hybrid architecture

The CNN-LSTM architecture is a composite neural network model that utilizes the capabilities of CNN to extract spatial characteristics from incoming data and employs LSTM networks to process sequential patterns within the data. Typically, this model begins by employing convolutional layers to pinpoint spatial patterns and subsequently passes these characteristics to LSTM layers to discern and model the temporal interrelations in the sequence (Davoudi and Roushangar, 2025). The typical architecture of CNN-LSTM models begins with a convolutional layer that extracts spatial features from the input information. The extracted features are subsequently fed into the subsequent LSTM layers, which handle the sequential relationships found in the data (Yang et al., 2021). The graphical architecture of the proposed hybrid CNN-LSTM model is shown in Figure 6. The proposed CNN-LSTM model comprises five key components arranged in a specific order for data processing. The layers were as follows: input, convolutional, pooling, LSTM, and dense. Following data pre-processing, the input layer subsequently receives the pre-processed input data, which are formatted in a structured manner that includes the time-step cycle, time-step dimension, and element factors. Subsequently, after the convolutional layer, the input data were processed by a dropout layer. The dropout mechanism randomly disables a specified percentage of neuron activation during forward propagation. The use of this technique is intended to prevent overfitting and offer a level of regulation to the model (Beresford et al., 2012). The hybrid CNN-LSTM model training process is described in Algorithm 1 and the overall federated CNN-LSTM framework is presented in Algorithm 2.

**FIGURE 6:**
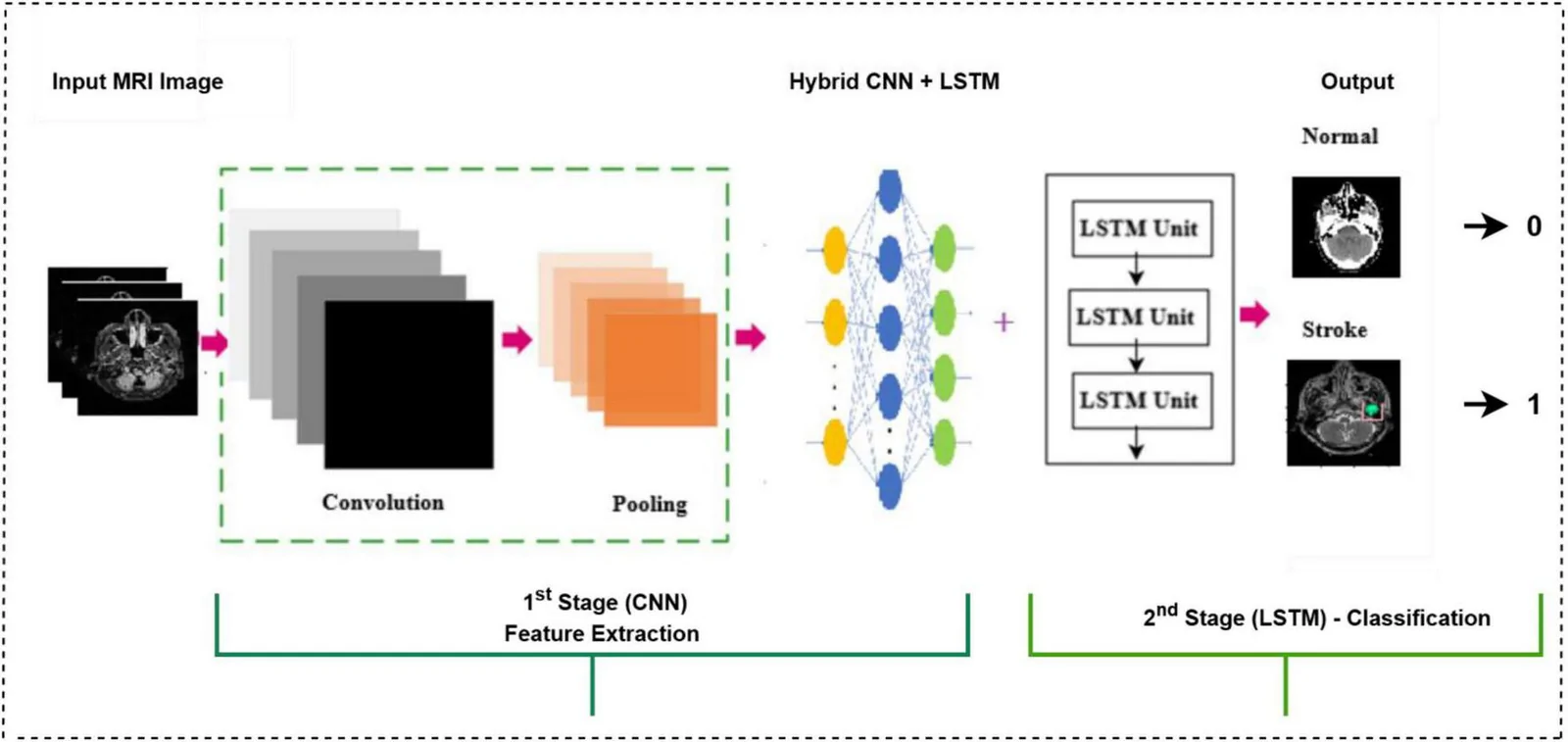
The organized hybrid’s architecture CNN-LSTM.

Algorithm 1: Hybrid CNN-LSTM

**procedure** HybridCNNLSTMPredict (InputSequence, CNN, LSTM, Classifier)

  Features ← []

  **for** each TimeStep in Input Sequence **do**

    Spatial Features ← CNN.extract_features (Timestep)

    Append Spatial Features to the Features

  **end for**

  Temporal Features ← LSTM.process_sequence (Features)

  Prediction ← Classifier.predict_class (Temporal Features)

  **return** Prediction

**end procedure**

Algorithm 2: Federated CNN-LSTM Framework

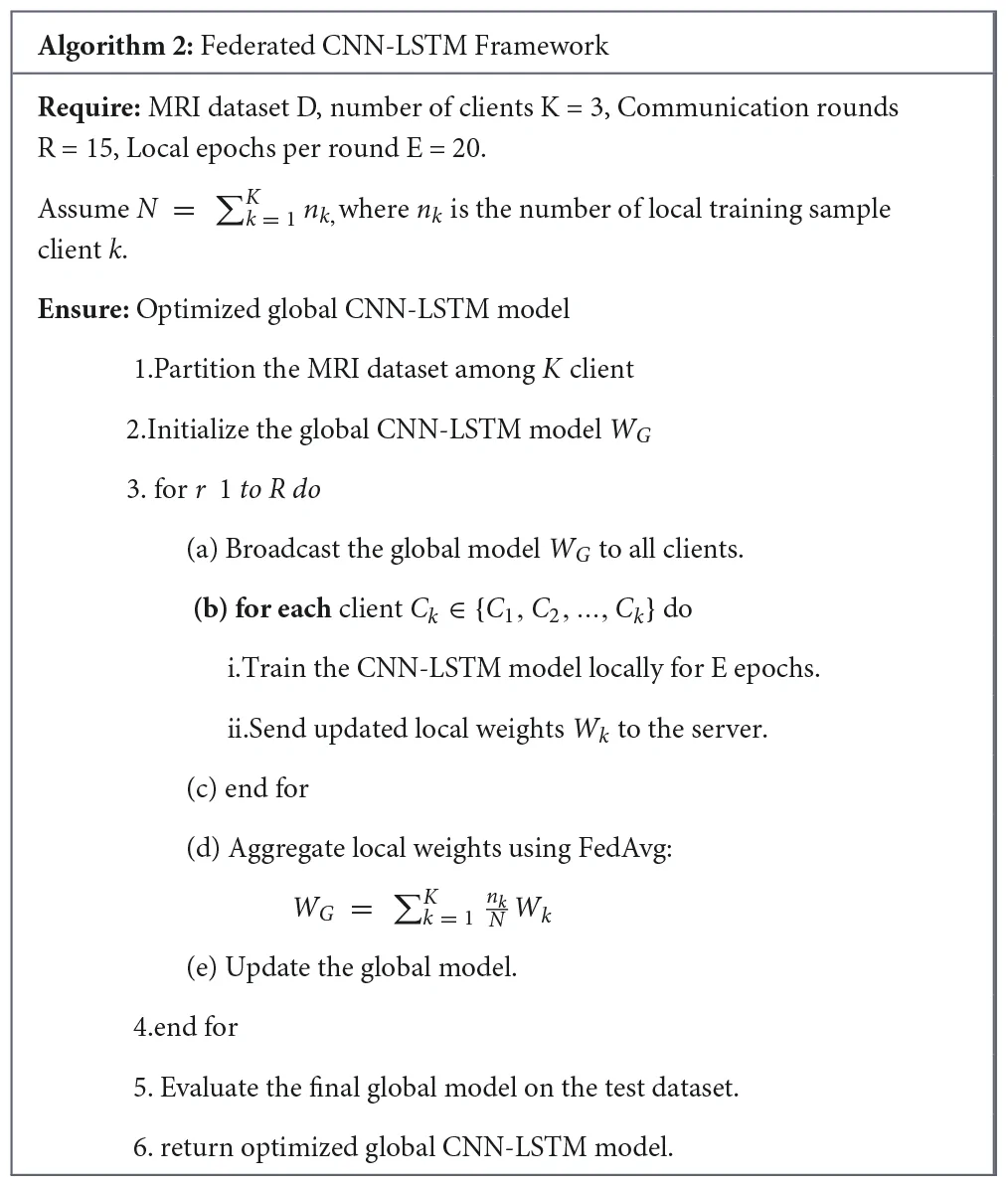

**Convolutional Neural Network:** The CNN, an extensively used reduction method initially developed, excels at recognizing local patterns in high-level input data and then feeding this information to subsequent layers to learn more complex features. Research on modeling spatiotemporal series with deep learning has primarily focused on recurrent neural network (RNN) architectures, such as LSTM and GRU; however, there is increasing interest among researchers in exploring the potential of convolutional networks as a primary method for analyzing time series data (Kavianpour et al., 2023). Figure 7 illustrates the structural design of the CNN.

**FIGURE 7:**
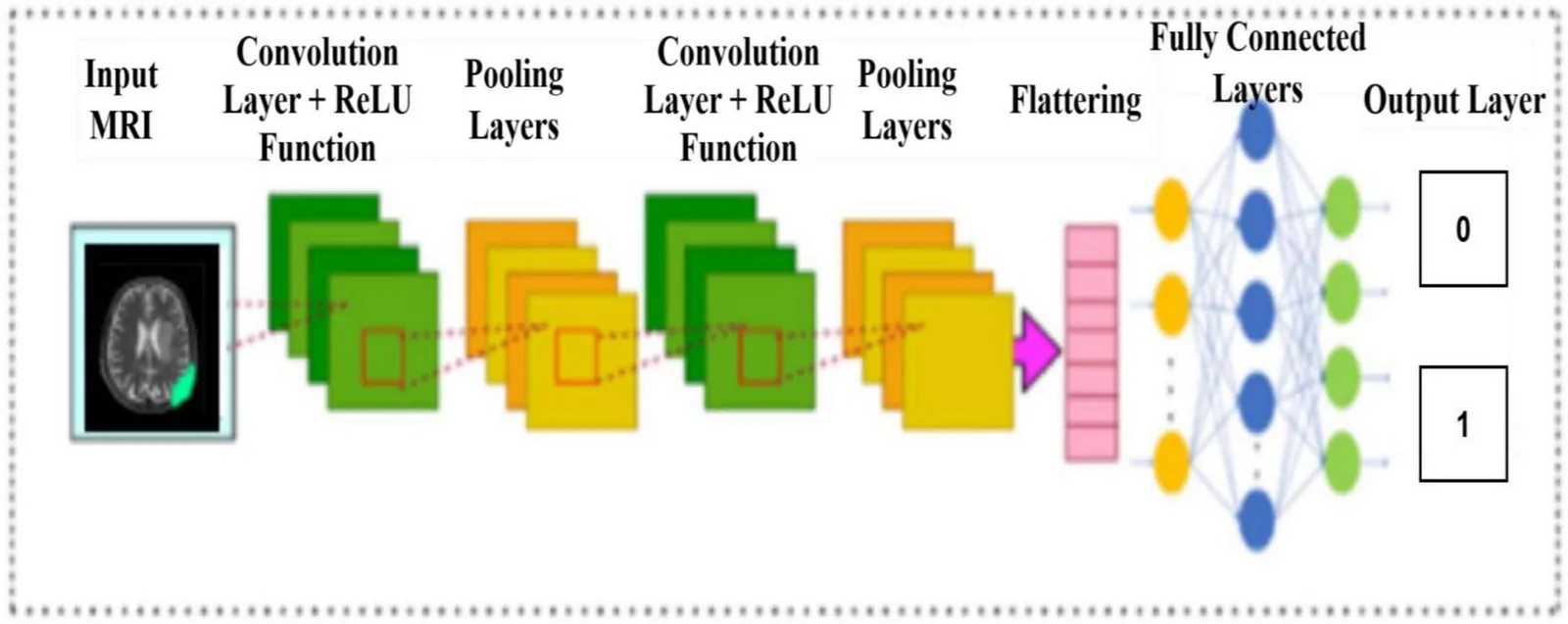
Architecture of the CNN.

**Long short-term memory**: The RNN, an advanced form of LSTM developed in 1997, differs from conventional feedforward networks. The main objective of the original design was to solve the persistent problem of the inability of typical RNNs to manage long-term dependencies. The original design’s main objective was to solve the persistent problem of typical RNNs’ inability to manage long-term dependencies. Furthermore, by adding memory cells, LSTM models address the issues of vanishing or exploding gradients that are frequently observed in RNNs. These memory cells are designed to store and retrieve information for a long time, which allows the neural network to gradually learn and recover patterns from a series of inputs (Synal and Wacker, 2010).

**Federated Learning:** A newly developed machine learning approach called Federated Learning addresses challenges stemming from stringent data privacy regulations and the limited availability of extensive datasets. In FL, a central server, referred to as a global server, exchanges information with numerous client-end systems or regional servers. At the core, a central server stores a global model, in contrast to individual client servers, which hold private local data stored on end-user devices. Model training can be performed using FL without the need to share actual data. The model is trained across many decentralized devices using data that are locally sourced from each device. The central server subsequently accumulates the revised model weights transmitted from each locally trained model. Preserving privacy is especially important in sensitive fields, such as medical data diagnosis (Zhang et al., 2021).

This study compared various hybrid CNN-LSTM architectures to determine the most effective one for developing a global model hosted on a central server. This asserts that the global model should not rely on any training data in accordance with the FL approach. A pretrained hybrid CNN-LSTM model was not used. Subsequently, local models were replicated, with each replica initialized with the same weight values as the master model. The client-server system simulated three clients, with the entire training dataset allocated among them. Three local models were then trained by their respective clients using stored data. Following local training, the model weights were consolidated, brought back, and normalized to a single group. Weight updates from the local models were sent back to revise the global model, which was subsequently tested with the validation data to diagnose brain strokes. The diagram below is used to visualize the FL model structure. The construction of the FL model is shown in Figure 8. Table 2 shows the hyperparameter settings of the proposed Federated CNN-LSTM model.

**FIGURE 8:**
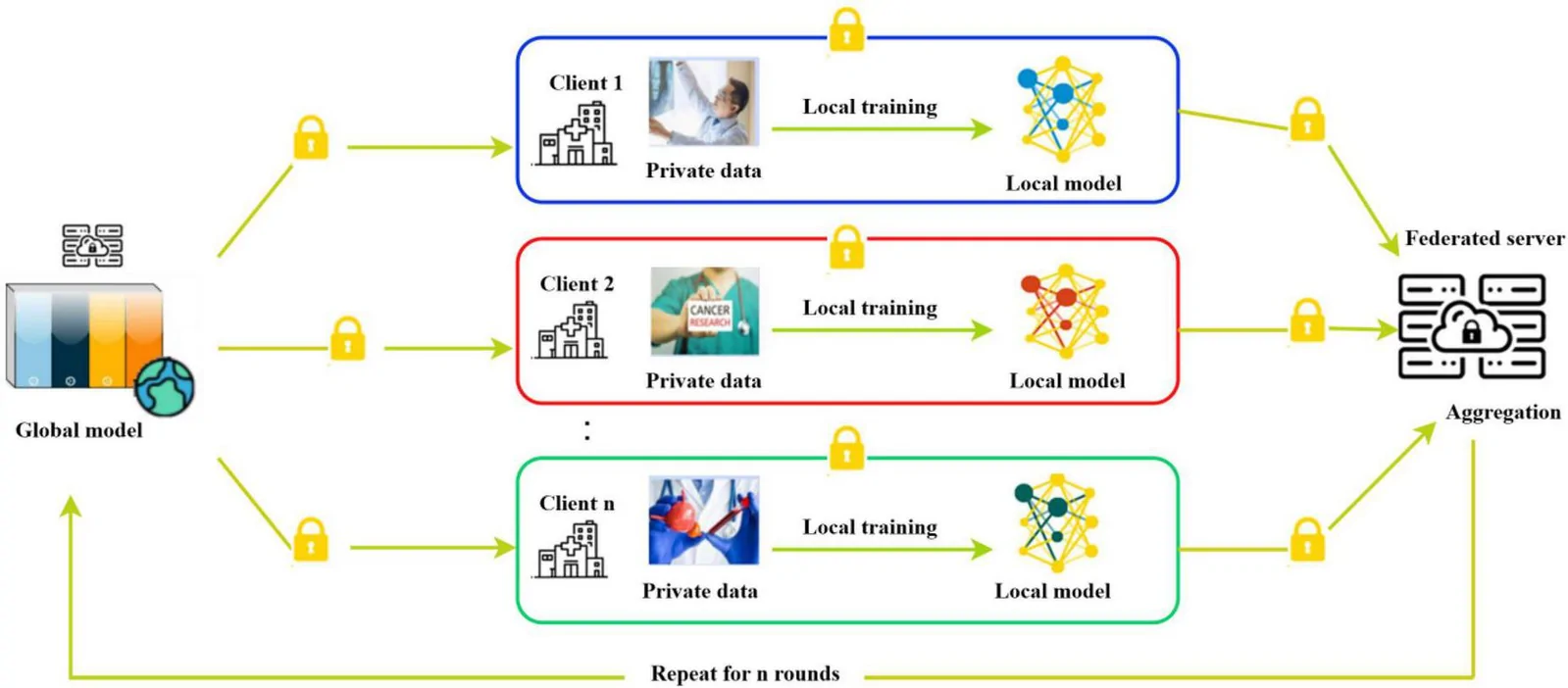
The Federated learning model structure

**TABLE 2:** Hyperparameter settings of the proposed Federated CNN-LSTM model.

Hyperparameter	Value
Image size	128 × 128 × 3
CNN layers	3 Conv blocks (32,64,128 filters)
LSTM hidden units	256
Optimizer	Adam
Learning rate	1e-4 (0.0001)
Batch size	16
Local epochs per round	20
Number of Clients	3
Communication rounds	15
Aggregation strategy	FedAvg
Cross-validation	Stratified 5-Fold
Class-weighted Cross-Entropy	[1.0, 1.5]

## 4 Experimental result analysis

The subsequent section presents the scores of the implementation, demonstrating the capabilities of the models in accurately recognizing brain strokes. Precision and recall were used to assess the classification performance, while the F1-score serves as a single numerical indicator of the system’s completeness. The metrics included precision, recall, F1-score, confusion matrix, accuracy, and loss function values for every model that was evaluated. The accuracy of the models in categorization during training depends on how well they can classify the training data. This essentially gauges the accuracy of the models in this context. Moreover, the loss function of a model is one of the most prominent and significant elements of a neural network with DL. This function signifies the extent to which the model’s predictions deviate from the actual results. By examining the model’s loss values, it can be inferred how poorly the model performs when making predictions from the dataset, such as predicting stroke. The test accuracy was assessed to evaluate the efficiency of the system. The primary goal was to achieve better test accuracy and reduce the model’s loss function value. The hybrid model was optimized over 20 iterations using Adam as the optimization algorithm with a learning rate of 0.0001. The work was effectively encapsulated by comparing it with other state-of-the-art techniques that have already been established and are being clinically explored. Axial 2D T2-weighted turbo spin echo (TSE) pictures from a 1.5 T scanner were included in a private dataset of MRI scans. Repetition time (TR) was set to 4620.0 ms, echo time (TE) to 105.0 ms, slice thickness to 5.0 mm, and in-plane resolution to 512 × 512 pixels. These photos initially represented 2D cross-sectional slices rather than 3D volumes and were saved in the DICOM format.

### 4.1 Performance evaluation of the hybrid CNN-LSTM model

The performance of the hybrid CNN–LSTM model is illustrated using the confusion matrix shown in Figure 9. The model correctly classified 293 instances as “Normal.” However, it misclassified 17 normal cases as “Stroke.” Additionally, 8 stroke cases were incorrectly predicted as “Normal.” On the other hand, the model successfully identified 307 stroke instances as true positives. Overall, the model achieved 600 correct predictions and 25 incorrect predictions. The accuracy is determined as the ratio of correctly classified instances to the total number of samples. Figure 10 illustrates the classification performance using a normalized confusion matrix expressed in percentages. The model accurately classified 94.52% of normal cases and 97.46% of stroke cases, with only a small margin of error (5.48% and 2.54%), demonstrating robust and well-balanced performance.

**FIGURE 9:**
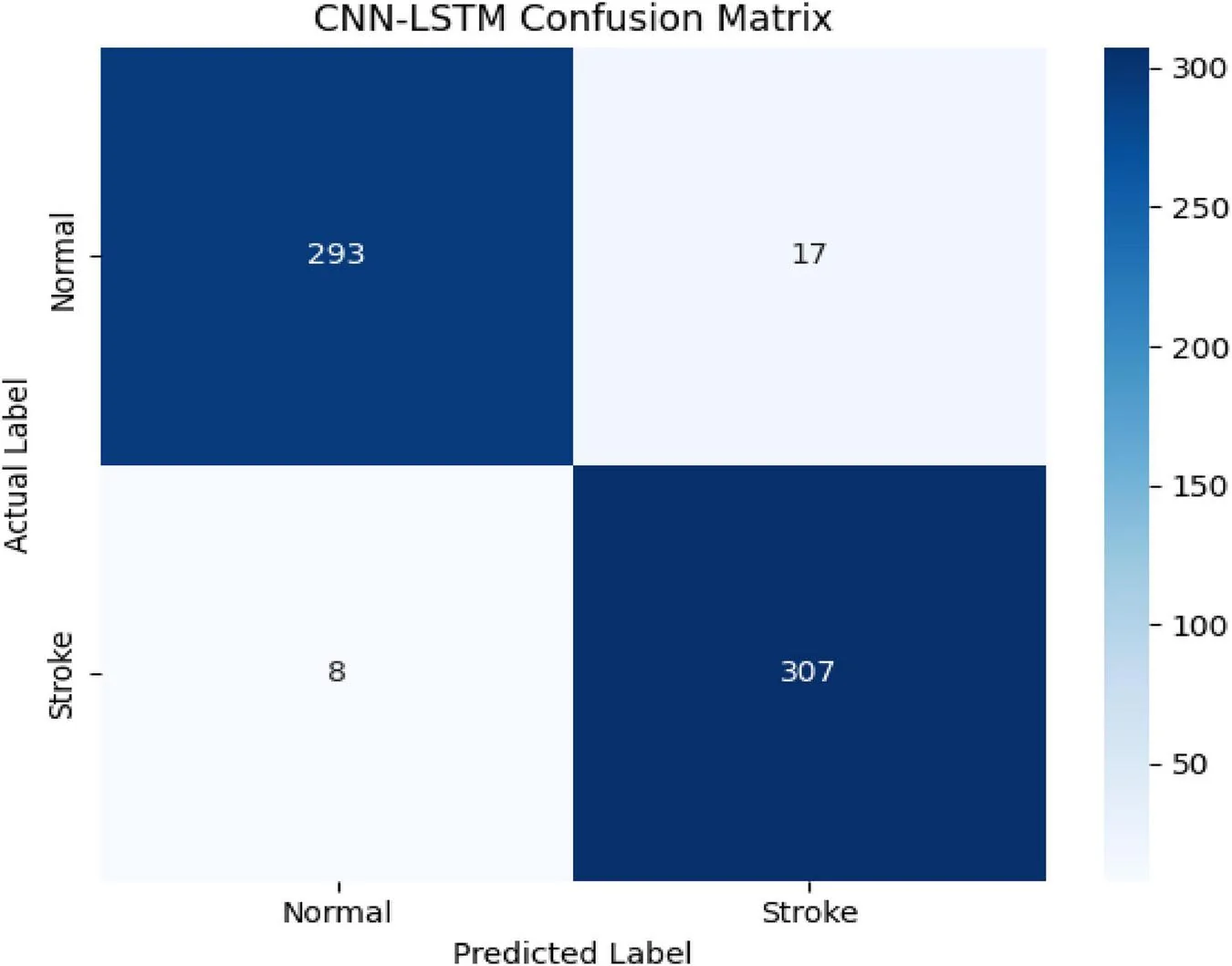
Confusion matrix of the hybrid CNN-LSTM model.

**FIGURE 10:**
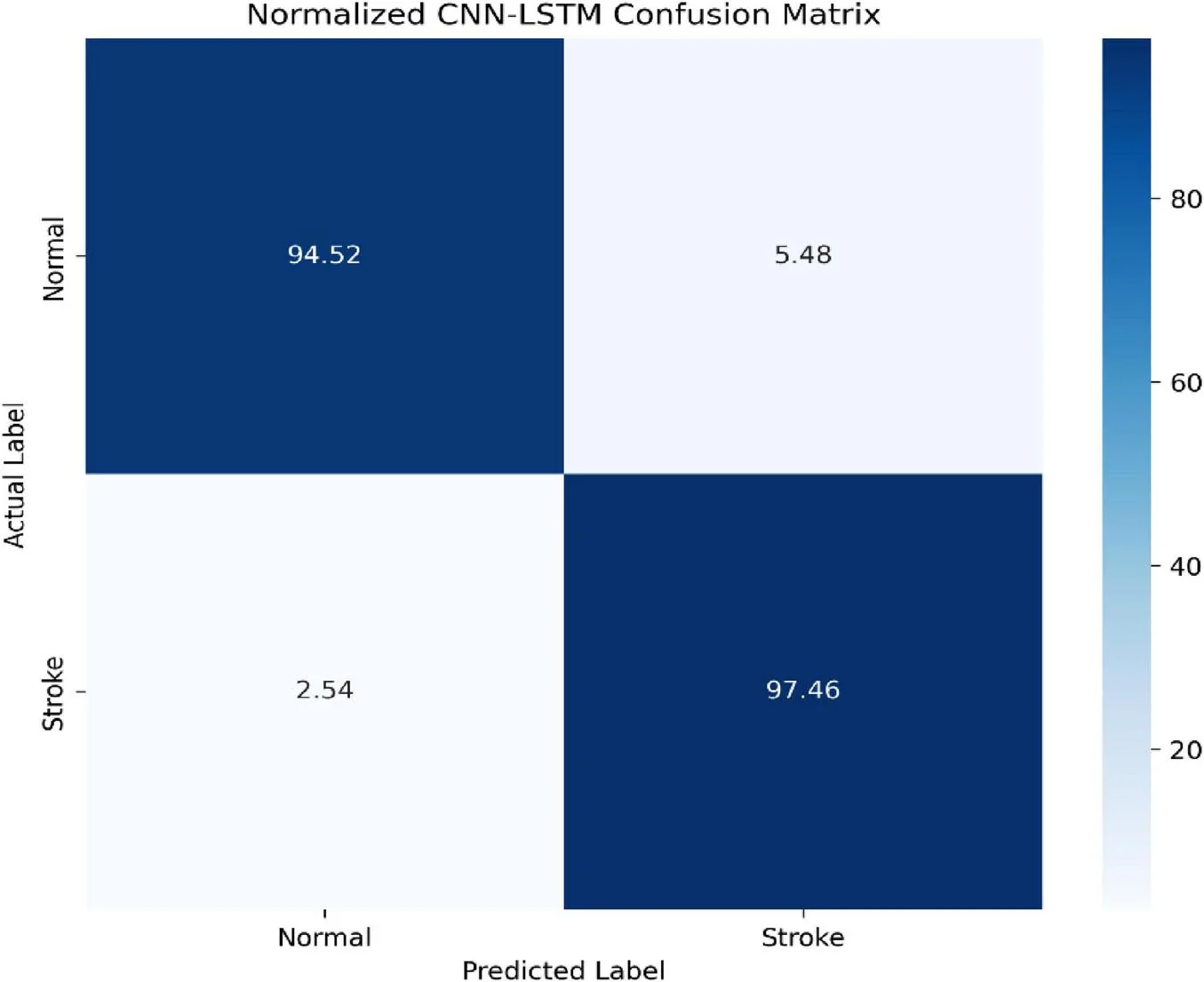
Normalized Confusion matrix of the hybrid CNN-LSTM model.

The performance of training and validation for the hybrid CNN-LSTM model is illustrated in Figure 11, which compares the preparation accuracy, confirmation exactness, and model loss function, demonstrating its superior performance. Additionally, Table 3 presents the performance metric values of the hybrid model, providing a detailed evaluation of its effectiveness. The performance analysis of the hybrid CNN-LSTM model is presented in Figure 12. The precision-recall (PR) curve is a graphical depiction of a model’s performance, particularly in binary classification problems. It plots the precision against the recall at different thresholds, providing a thorough understanding of the model’s capacity to identify positive examples. A high recall indicates that the model is successful in identifying positive cases, whereas a high precision indicates that the model is accurate in its predictions. Figure 13 shows the precision-recall curve. The PR curve helps identify the optimal threshold for the model, balancing precision and recall.

**FIGURE 11:**
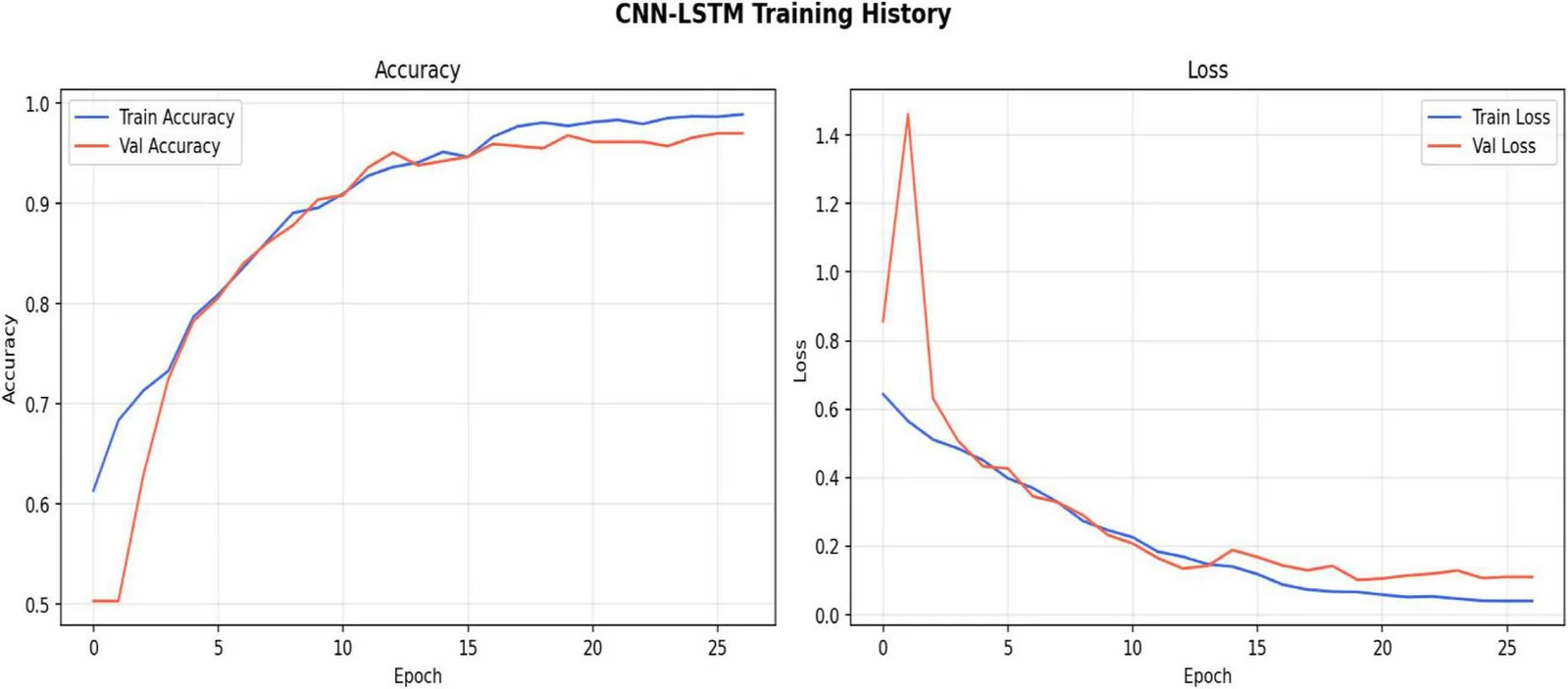
Training and Validation ACC and loss function of the hybrid CNN-LSTM model.

**TABLE 3:** Classification report for the CNN-LSTM hybrid model.

Metrics	Prec	Recall	F1-Score	Acc	Sens	Spec
Values	0.9599	0.9594	0.9595	0.9595	0.9746	0.9442

**FIGURE 12:**
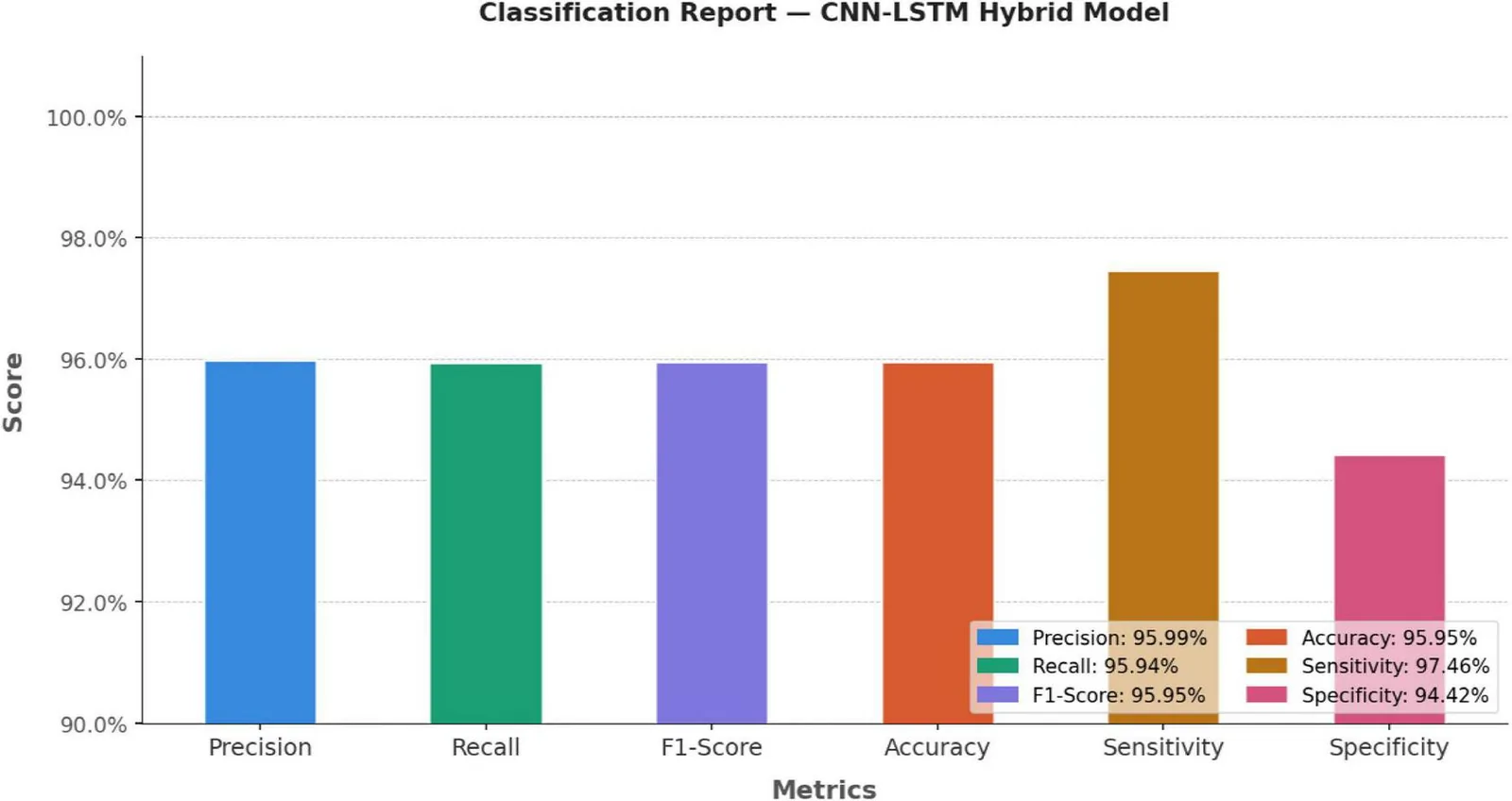
Classification performance of the hybrid CNN-LSTM model.

**FIGURE 13:**
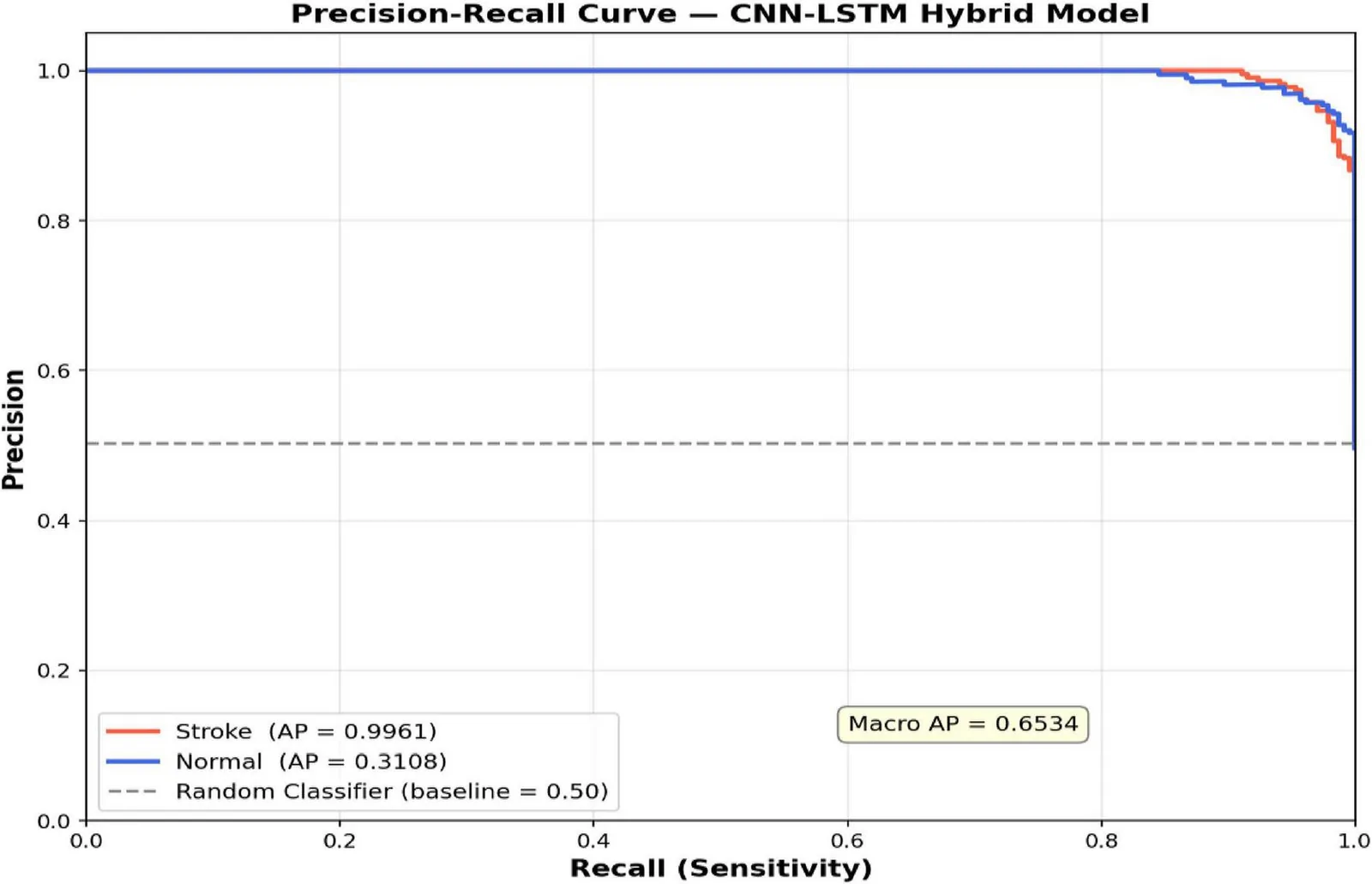
Hybrid CNN-LSTM model Precision-Recall curve.

The outcome of a model for binary classification problems can be observed schematically by the Receiver Operating Characteristic (ROC) curve, which depicts the True Positive Rate (TPR) versus the False Positive Rate (FPR) across multiple thresholds. A good classifier has a superior TPR and low FPR, correctly identifying positive instances while minimizing false alarms. The area under the ROC curve (AUC-ROC) represents the model’s overall performance, with higher rates indicating better performance. The ROC curve is useful for evaluating and comparing the performance of binary classification models, identifying the optimal threshold, and making informed decisions. By analyzing the ROC curve, preserve acquired perceptions of a pattern’s strengths and weaknesses, and adjust to improve its performance. Figure 14 shows the ROC curve of the hybrid model.

**FIGURE 14:**
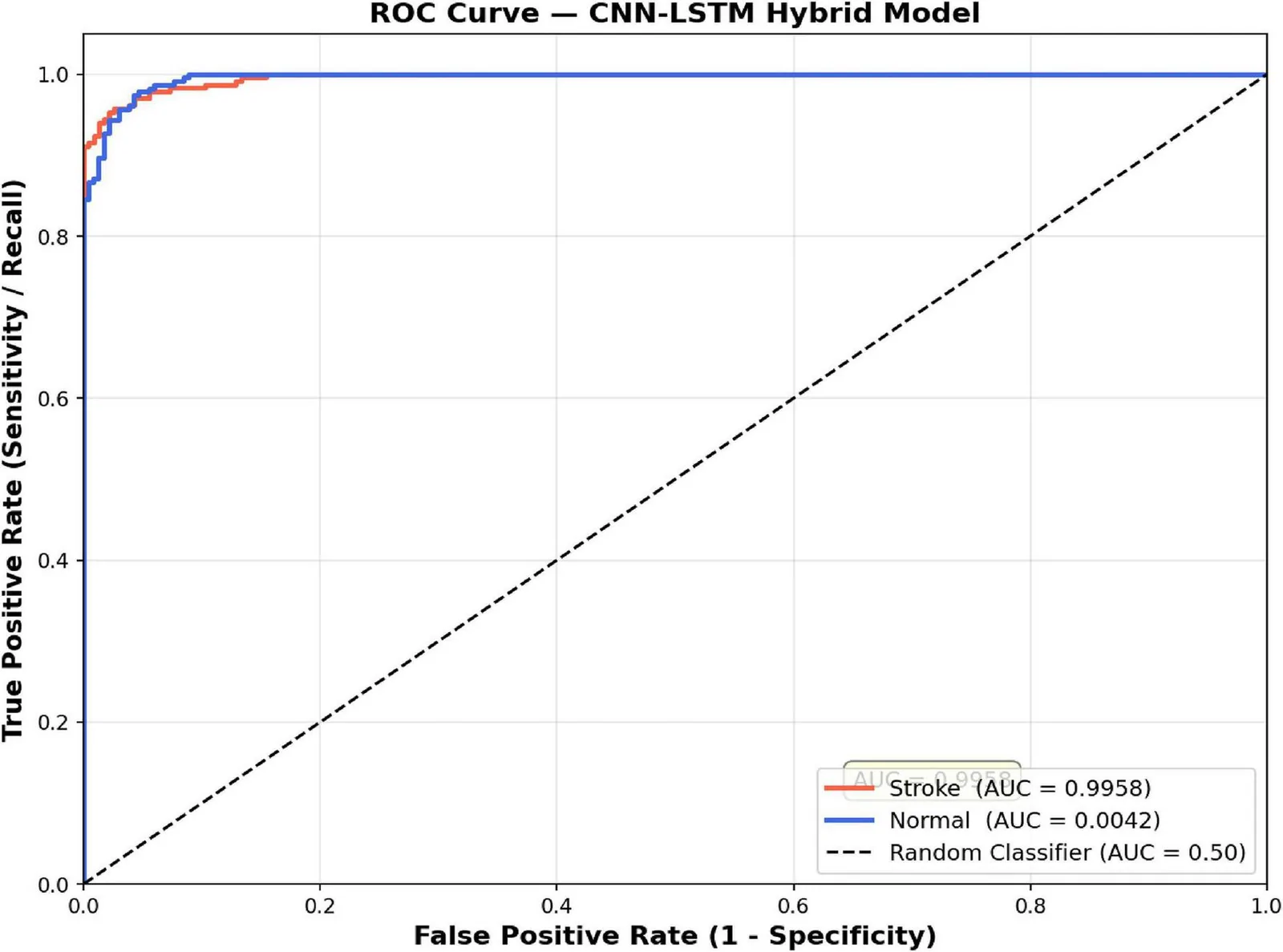
ROC curve of hybrid CNN-LSTM model.

### 4.2 Performance evaluation of federated learning

Due to its exceptional performance, the hybrid CNN–LSTM architecture was employed to create a global model suitable for the Federated Learning (FL) framework. Subsequently, three local models were initialized with the weights of the global model and distributed among three clients. The entire dataset comprised 3125 samples, which were split into 2500 training samples and 625 testing samples. The complete dataset was distributed among clients in a highly non-IID manner, with significant variation in class proportions. Specifically, Client 1 contained 800 normal and 100 stroke samples, Client 2 contained 100 normal and 900 stroke samples, and Client 3 contained 651 normal and 574 stroke samples. This heterogeneous distribution reflects real-world scenarios where data is unevenly distributed across clients. In total, the Non-IID distributed dataset for FL consisted of 1551 normal and 1574 stroke samples, as presented in Table 4. To address this imbalance and ensure equitable contributions from each client, a scaling factor was applied, as specified in Equation (1), and the precision of each local model was assessed. The scaled local weights were then combined to update the global model.

**TABLE 4:** Non-IID data distribution across FL clients.

Client	Normal	Stroke
Client 1	800	100
Client 2	100	900
Client 3	651	574
Total	1551	1574

To mitigate overfitting and improve training stability, image intensity normalization to the [0, 1] range and batch normalization were employed. The CNN-LSTM architecture also incorporated dropout layers in the centralized model, while the federated CNN-LSTM model used batch normalization. Five-fold stratified cross-validation was conducted to evaluate model robustness and generalizability. The federated training was performed using the FedAvg algorithm over 15 communication rounds for the main experiment, with a learning rate of 1 × 10^−4^ and three local training epochs per round. Model performance was evaluated using accuracy, precision, recall, F1-score, ROC-AUC, and precision-recall curves.


$$scalingcomponent\left(s.c\right)=\frac{a\,m\,o\,u\,n\,t\,o\,f\,c\,l\,i\,e\,n\,t\,d\,a\,t\,a}{a\,m\,o\,u\,n\,t\,o\,f\,t\,o\,t\,a\,l\,d\,a\,t\,a}$$ (1)

A limitation of the proposed model is that it only reveals a small amount of data information, as the quantity of images per client can be assessed. However, the core principle of FL was maintained, and no information was transmitted to the global model. Instead, the weights of the individual local models were scaled using a scaling factor, as shown in Equation (2), and stored in a variable, thus preserving data privacy.


s⁢c⁢a⁢l⁢e⁢d⁢l⁢o⁢c⁢a⁢l⁢w⁢e⁢i⁢g⁢h⁢t⁢s=s.c⁢X⁢l⁢o⁢c⁢a⁢l⁢m⁢o⁢d⁢e⁢s′⁢w⁢e⁢i⁢g⁢h⁢t (2)

In this FL setup, the weights of the three clients were scaled, aggregated, and averaged, and then operated to modernize the weights of the global model on a central server. This process was repeated for 15 communication rounds, with the accuracy of the global model in detecting brain stroke evaluated on a test dataset after each round. Each client trained its model for 10 epochs, with a batch size of 16 and a learning rate of 0.0001. This approach enables shared learning, allowing the global model to benefit from the collective knowledge of multiple clients, intending to improve brain stroke detection performance. The evaluation criteria for this model are as follows. The confusion matrix offers a straightforward depiction of the model’s effectiveness. It recorded 284 true negatives, indicating that the model accurately identified the “normal” cases. There were eight false negatives, in which stroke cases were mistakenly predicted as normal, and 22 false positives, in which normal cases were wrongly classified as stroke. Conversely, the model successfully recognized 311 true positive stroke cases. In total, the model made 595 correct predictions (284 + 311) and 30 incorrect predictions (22 + 8) out of 625 samples. Communication cost and training time analysis of the federated CNN-LSTM model is shown in Table 5.

**TABLE 5:** Communication cost and training time analysis of the federated CNN-LSTM model.

Category	Parameter	Value
Communication Cost	Number of Clients	3
	Model Size (FP32)	0.1304 MB
	Total Communication, 15 rounds (FP32)	11.736 MB
	Total Communication, 15 rounds (FP16)	5.868 MB
	FP16 reduction	2 × (50%)
Training Time	Total FL training time(sequential)	585.93 s
	Avg. time per round	39.06 s
	Theoretical parallel time (15 rounds)	∼197.65 s
	Time saved with parallel clients	∼66.2%

The model’s accuracy was determined by dividing the number of correct classifications by the total number of samples. The model’s performance was assessed using the confusion matrix, as shown in Figure 15. The performance metrics of the CNN-LSTM-FL model are presented in Table 6, which offers a comprehensive assessment of its effectiveness.

**FIGURE 15:**
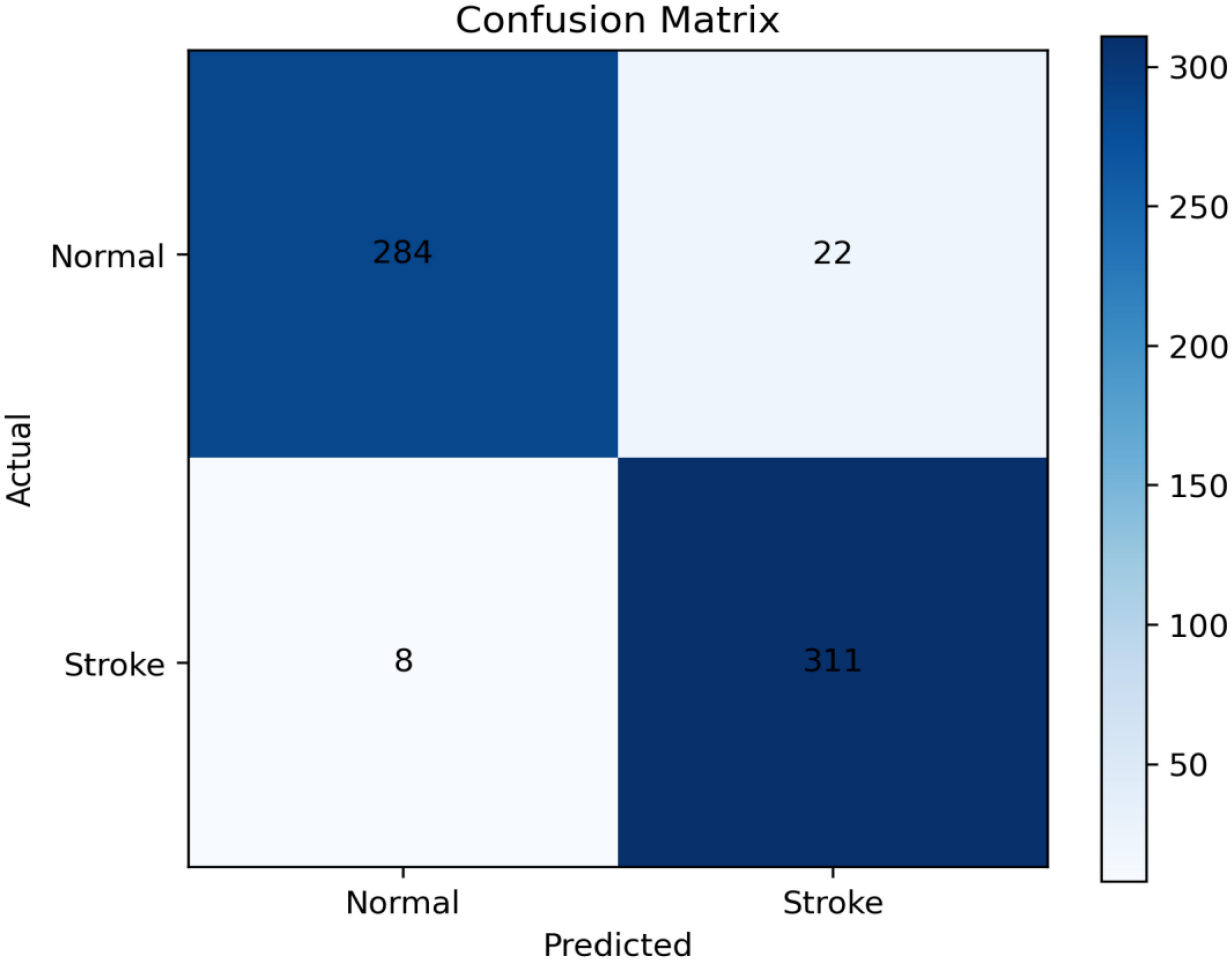
Confusion matrix of the federated learning.

**TABLE 6:** Report on the FL model’s classification.

Metrics	Prec	Recall	F1-Score	Acc	Sens	Spec
**Values**	0.9341	0.9750	0.9543	0.9520	0.9743	0.9281

Figure 16 shows the model’s accuracy function. Figure 17 shows the efficiency metrics of the CNN-LSTM-FL model, including accuracy, precision, recall, and F1-score. The Receiver Operating Characteristic (ROC) curve was used to assess and compare the effectiveness of binary classification models, identify the optimal threshold, and facilitate well-informed decision-making. Analyzing the ROC curve can help understand a model’s capabilities and shortcomings, allowing for the adjustment and enhancement of its overall performance. Figure 18 shows the ROC curve for the FL model. Figure 19 shows the precision-recall curve of the CNN-LSTM-FL model. The Grad-CAM visualization highlights the brain MRI regions that contribute most to the model’s prediction. The red and yellow areas indicate close attention, showing that the model focuses on relevant anatomical regions associated with stroke, whereas the blue regions have minimal influence on the decision. Figure 20 illustrates the Grad-CAM visualization.

**FIGURE 16:**
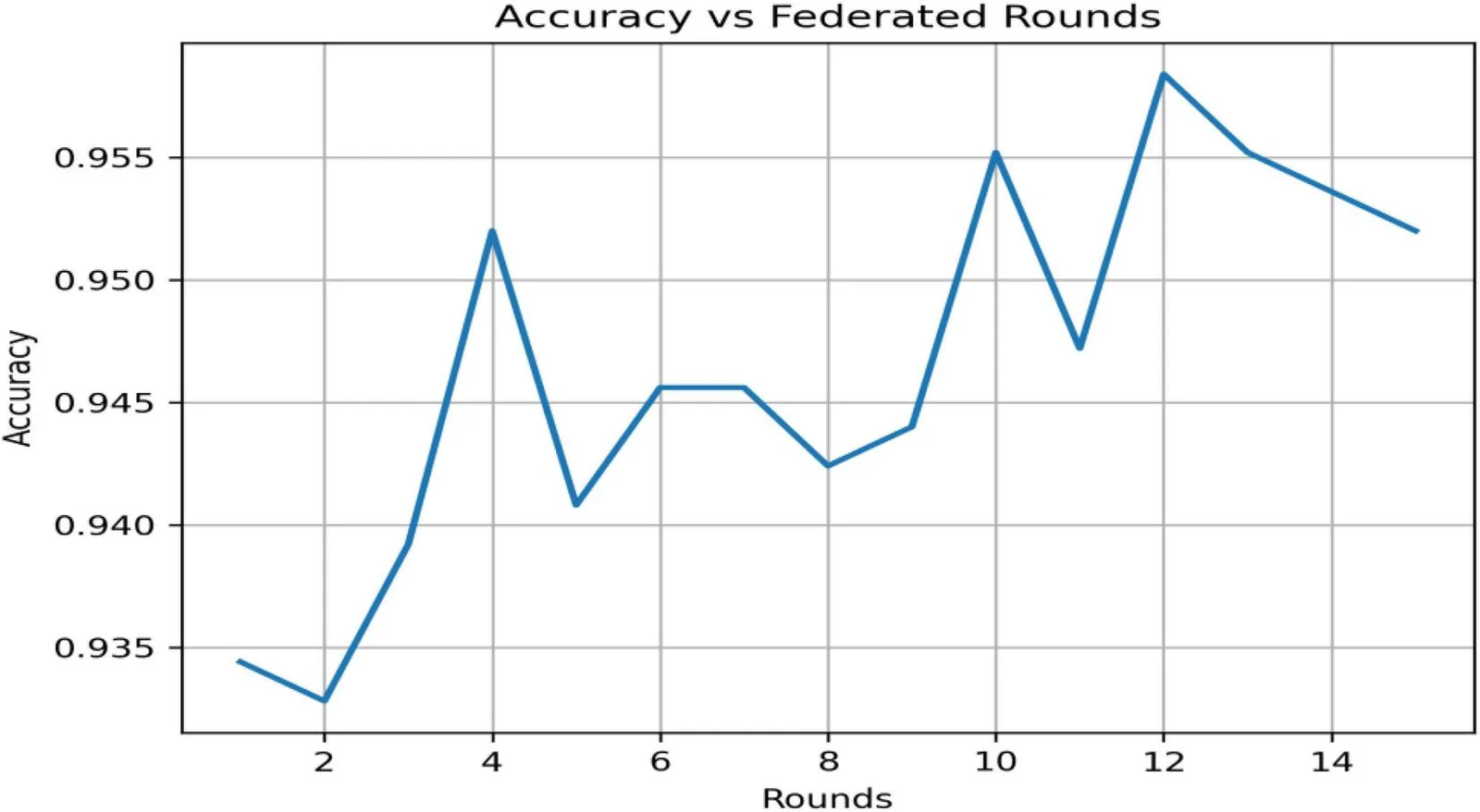
Accuracy of the federated learning model.

**FIGURE 17:**
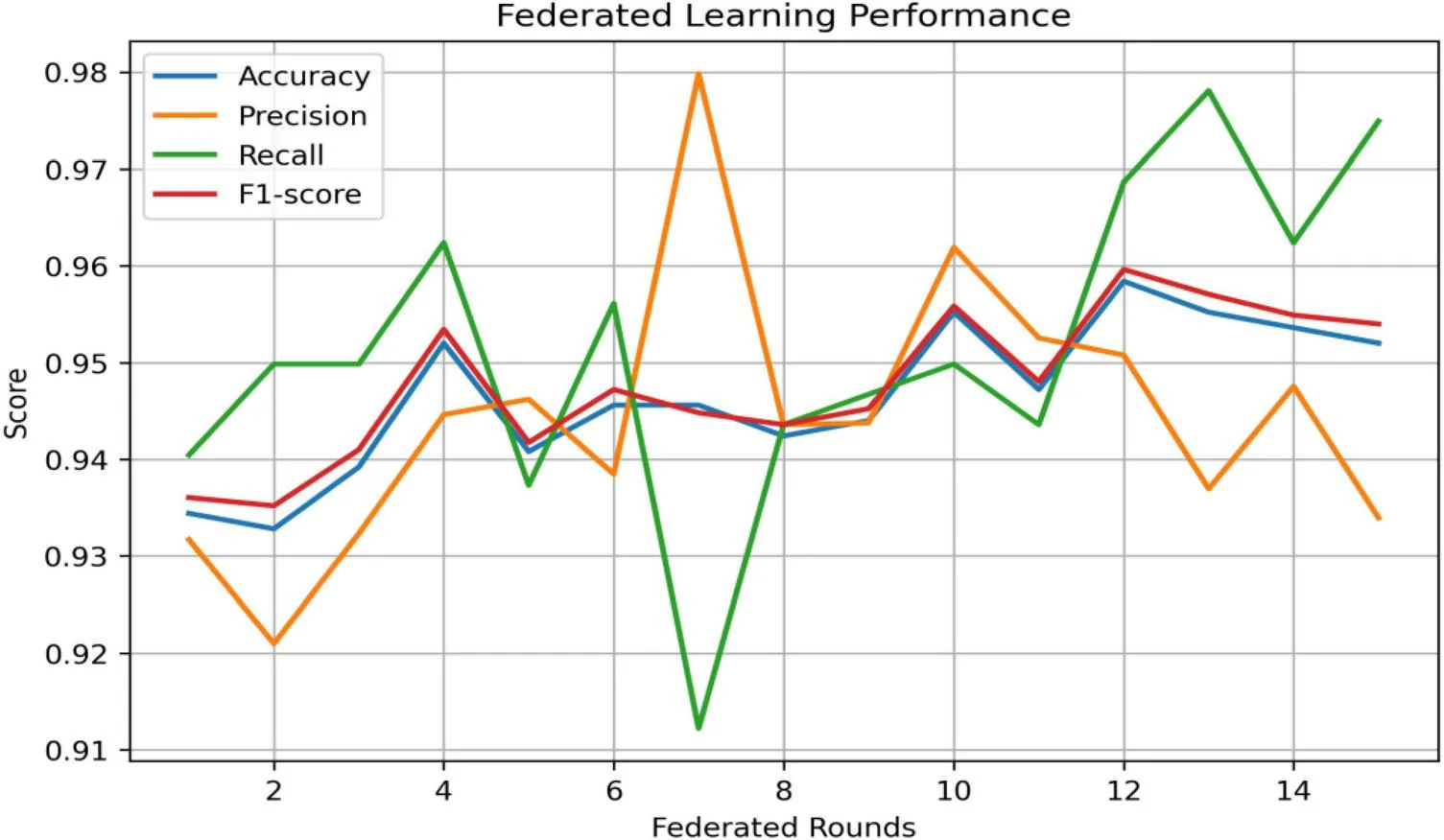
Evaluation of the hybrid CNN-LSTM with federated learning model’s accuracy, precision, recall, and F1-score.

**FIGURE 18:**
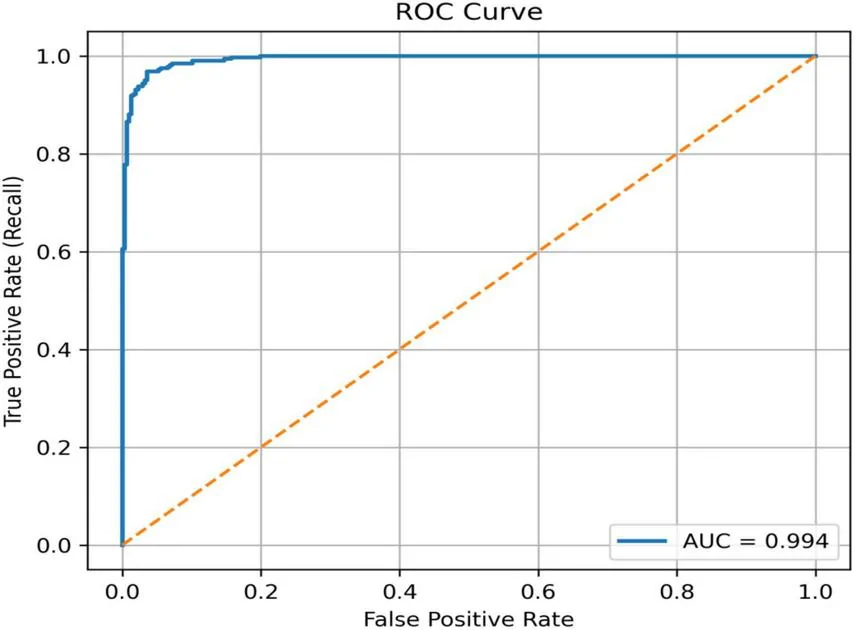
ROC curve of CNN-LSTM-FL model.

**FIGURE 19:**
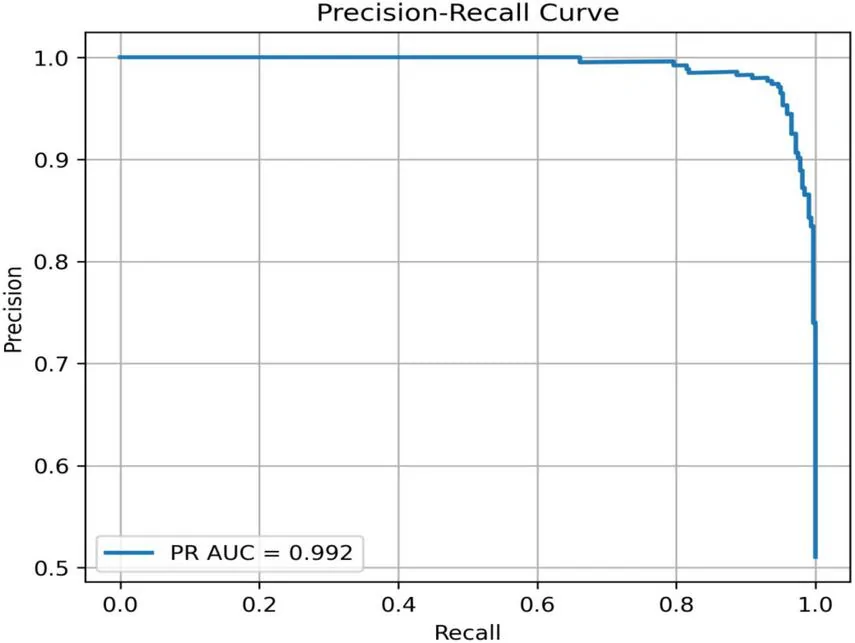
Precision-Recall curve of CNN-LSTM-FL model.

**FIGURE 20:**
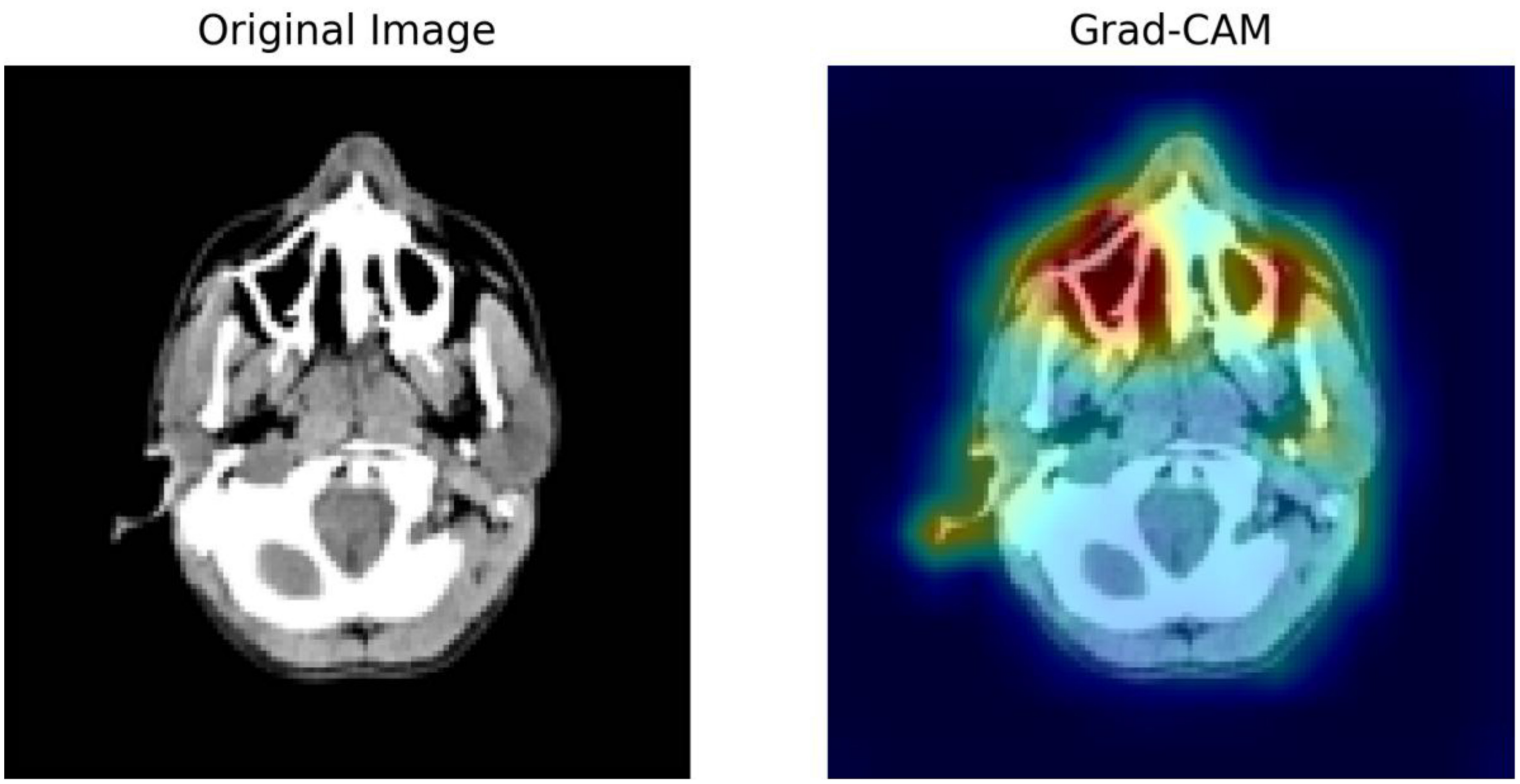
Interpretability analysis using Grad-CAM to highlight important regions influencing the model’s prediction.

### 4.3 Analogy with state-of-the-art techniques

Table 7 presents a classification report comparing the performance of the four models: CNN, ResNet50, Hybrid CNN-LSTM, and federated learning. The metrics used for the comparison were precision, recall, and F1-score. The results showed that the hybrid CNN-LSTM model achieved superior precision, recall, and F1-score values, representing strong performance. The hybrid CNN-LSTM- FL also showed promising results, with precision, recall, and F1-score values of 0.93, 0.97, and 0.95, respectively. Figure 21 illustrates the overall accuracy comparison between the proposed method and state-of-the-art approaches using the MRI dataset.

**TABLE 7:** Comparative classification performance of four models.

Model	Prec	Recall	F1-Score
CNN	0.92	0.89	0.94
ResNet50	0.48	0.47	0.48
LSTM	0.86	0.87	0.90
Hybrid CNN-LSTM	0.96	0.95	0.95
Proposed	0.93	0.97	0.95

**FIGURE 21:**
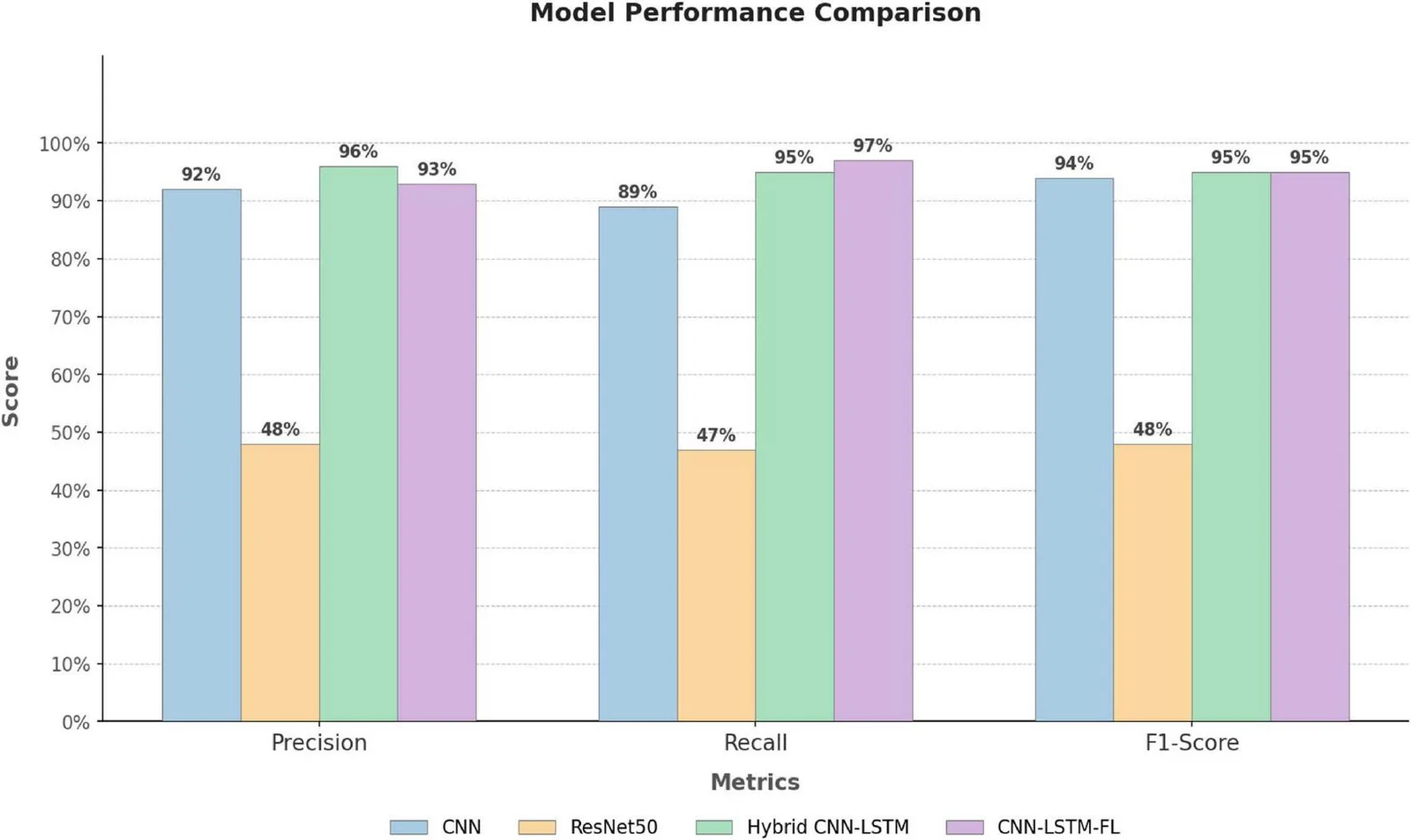
Overall comparison among the proposed models.

## 5 Ablation study

A 5-fold cross-validation experiment was carried out to assess the stability and generalization of the suggested federated CNN-LSTM framework. The cross-validation results for each of the five folds are compiled in Table 8. With an average accuracy of 92.10% ± 1.50% and a mean F1-score of 92.20% ± 1.36%, the model showed strong and consistent performance. The model’s high sensitivity in identifying stroke cases – a critical requirement in clinical diagnostic settings to reduce missed diagnoses – was demonstrated by its noteworthy mean recall of 92.50% ± 1.26%. The robustness and stability of the federated framework under different data distributions are confirmed by the low standard deviation seen across all evaluation metrics. Figure 22 shows the five-fold cross-validation for the federated CNN-LSTM model.

**TABLE 8:** Five-fold cross-validation results of the Federated CNN-LSTM model.

Fold	Accuracy	Precision	Recall	F1-Score
1	0.9200	0.9102	0.9333	0.9216
2	0.8944	0.8694	0.9302	0.8988
3	0.9376	0.9395	0.9365	0.9380
4	0.9328	0.9417	0.9238	0.9327
5	0.9200	0.9371	0.9013	0.9188
Mean	0.9210	0.9196	0.9250	0.9220
std	0.0150	0.0276	0.0126	0.0136
95% CI	[0.9024, 0.9396]	[0.8853, 0.9539]	[0.9094, 0.9406]	[0.9051, 0.9389]

**FIGURE 22:**
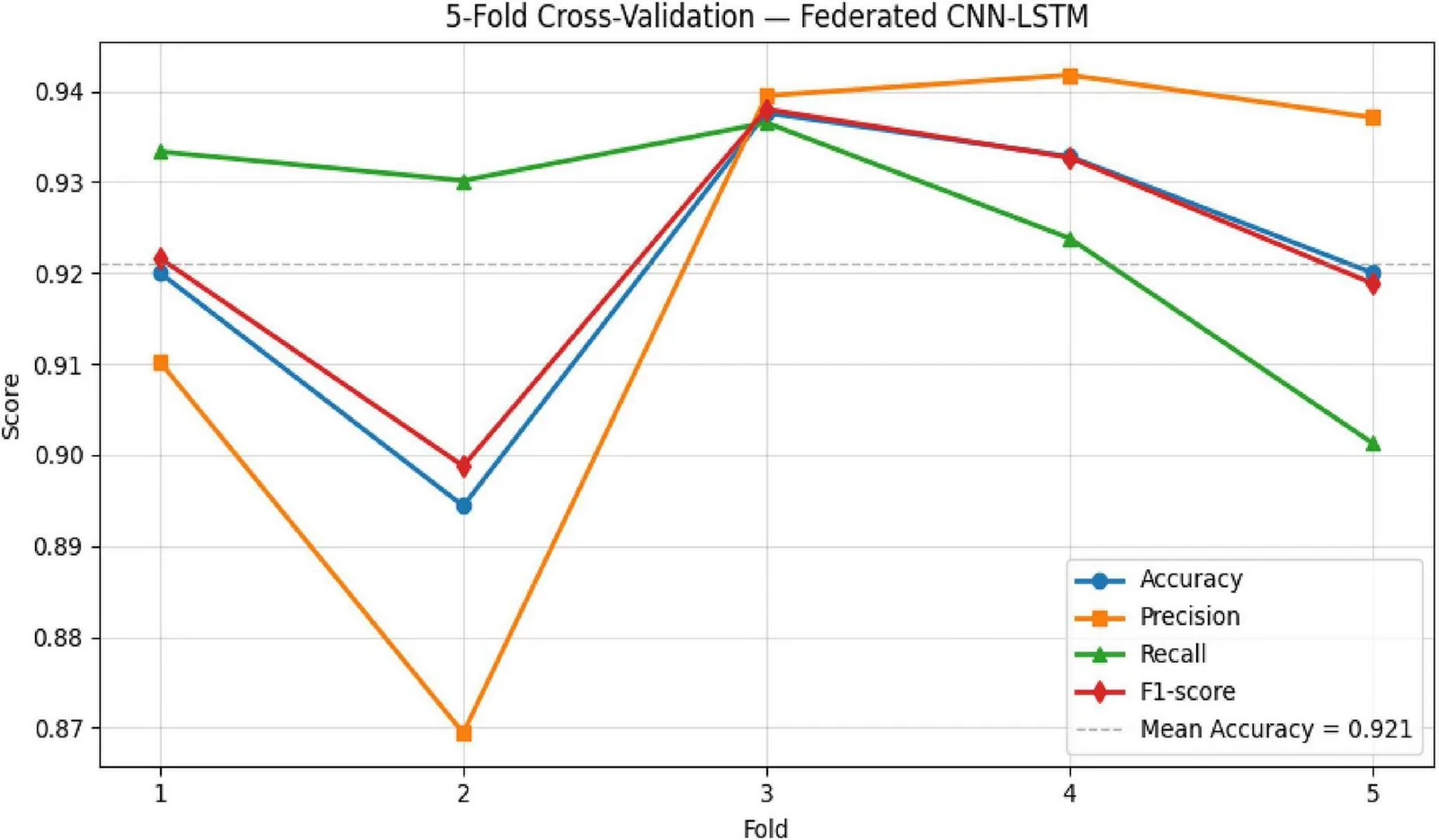
Five-Fold Cross Validation for Federated CNN-LSTM Model.

To assess the statistical reliability of the reported results, 95% confidence intervals (CIs) were computed for each performance metric across the five folds using the t-distribution (df = 4, t = 2.776), given the small sample size (*n* = 5). The Federated CNN-LSTM model achieved a mean accuracy of 92.10% (95% CI: 90.24%–93.96%), precision of 91.96% (95% CI: 88.53%–95.39%), recall of 92.50% (95% CI: 90.94%–94.06%), and F1-score of 92.20% (95% CI: 90.51%–93.89%), confirming that the model’s performance was statistically stable and consistent across data partitions.

To assess each component’s contribution, the ablation study contrasts the basic models with other architectural configurations. BiLSTM-Only outperforms LSTM-Only in terms of accuracy among the individual sequence models (88.60% vs. 87.30%). With 95.95% accuracy, 95.99% precision, 95.94% recall, and a 95.95% F1-score, the suggested hybrid CNN-LSTM, however, performs the best overall. Additionally, it only needs 1.8 million parameters, indicating a satisfactory trade-off between model complexity and performance.

CNN-LSTM-FL attains an accuracy of 95.20%, precision of 93.41%, recall of 97.50%, and F1 score of 95.43%. It demonstrates that federated training retains performance levels comparable to the centralized hybrid architecture. While inference and parameters have a slight increase, the major benefit provided by this architecture is privacy preservation during collaborative learning. The findings clearly demonstrate that the combination of CNN for spatial feature extraction and LSTM for sequential learning greatly enhances classification performance levels. The ablation study carried out to evaluate the influence of every element in the proposed model is shown in Table 9.

**TABLE 9:** Ablation study results: component-wise performance analysis.

Configuration	Acc (%)	Prec (%)	Rec (%)	F1-Score (%)
CNN (Baseline)	90.40	92.10	89.12	94.10
ResNet50	50.20	48.50	47.80	48.10
VGG16	87.80	86.90	88.20	87.50
Ablation configuration				
a) LSTM-Only	87.30	86.80	87.70	90.20
b) BiLSTM-Only	88.60	87.40	89.10	88.20
c) Hybrid CNN-LSTM	95.95	95.99	95.94	95.95
d) CNN-LSTM-FL	95.20	93.41	97.50	95.43

## 6 Conclusion and future work

This study addressed the critical challenge of limited access to medical diagnostic datasets, which are often restricted by privacy regulations, by proposing a privacy-preserving framework for brain stroke classification. A hybrid convolutional neural network–long short-term memory (CNN-LSTM) architecture was employed in conjunction with FL to analyze MRI images in a decentralized manner. To enhance the model’s robustness and mitigate the risk of overfitting on constrained datasets, rigorous data augmentation techniques were applied during preprocessing. Furthermore, to ensure the clinical interpretability of the network, gradient-weighted class activation mapping (Grad-CAM) was utilized to visualize and validate the localized regions of interest that drive the model’s predictions. The hybrid CNN-LSTM model achieved a high classification accuracy of 95.95% in a centralized setting. Crucially, the FL-based approach successfully maintained patient data privacy while achieving a commendable accuracy of 95.20%. The Federated CNN-LSTM model exhibited optimized generalization abilities, reaching an average accuracy of 92.10% during 5-fold cross-validation. The small standard deviation of 0.0150 suggests that its performance was stable and consistent across various folds. These results demonstrate that FL can be effectively integrated with advanced deep learning architectures and explainability tools to enable secure, transparent, and highly accurate decentralized medical image analysis.

## 7 Limitations

- The dataset used in this study was relatively small. It may not fully capture variations in stroke characteristics, imaging protocols, scanner types, and patient demographics, which may affect the generalizability of the model.
- DICOM MRI images were converted to JPEG images. Although this aids computation, minor information loss during conversion could affect fine features.
- Splitting in the given dataset has not been done based on patient identifiers; however, image splitting has been performed. This may cause some potential data leakage, which could lead to an optimistic estimation in the future.
- The model’s generalizability may be restricted as the dataset is sourced from a single hospital without external and cross-institutional validation.
- Although promising, the performance of the model using FL showed a notable accuracy drop compared to the centralized model.
- Implementing federated learning is more time-consuming than using hybrid models.
- Communication overhead and privacy risks such as gradient leakage were not explicitly evaluated in the present study.

## 8 Future work

✓ The evaluation of the hybrid CNN-LSTM model with FL should be extended to diverse and larger MRI datasets across different institutions to assess generalizability
✓ Improved FL aggregation algorithms should be developed to enhance global model accuracy and stability.
✓ External validation to confirm the model’s robustness across multi-center datasets, potentially utilizing privacy-preserving federated learning frameworks to securely manage diverse clinical data.
✓ Advanced feature extraction and image pre-processing techniques should be explored to handle low-quality or inconsistent MRI images more effectively.
✓ The application of the proposed approach to other medical imaging tasks beyond stroke classification should be examined to validate its broader applicability.
✓ Addressed vulnerabilities like gradient leakage and proposed differential privacy as a future safeguard.
✓ Explore 3D CNNs and Vision Transformer (ViT) for improved MRI feature extraction and stroke detection.

## Data Availability

The raw data supporting the conclusions of this article will be made available by the authors, without undue reservation.
